# Impact of contextual factors on patient outcomes following conservative low back pain treatment: systematic review

**DOI:** 10.1186/s12998-022-00430-8

**Published:** 2022-04-21

**Authors:** Bronwyn Sherriff, Carol Clark, Clare Killingback, Dave Newell

**Affiliations:** 1 Department of Rehabilitation and Sport Sciences, Faculty of Health and Social Sciences, Bournemouth University, Bournemouth, England; 2 AECC University College, Bournemouth, England; 3 Department of Sport, Health and Exercise Sciences, Faculty of Health Sciences, University of Hull, Hull, England

**Keywords:** Contextual factors, Placebo effect, Chronic low back pain, Illness beliefs, Communication, Verbal suggestion, Physician–patient relations, Empathy, Therapeutic alliance

## Abstract

**Background and objective:**

Chronic low back pain is pervasive, societally impactful, and current treatments only provide moderate relief. Exploring whether therapeutic elements, either unrecognised or perceived as implicit within clinical encounters, are acknowledged and deliberately targeted may improve treatment efficacy. Contextual factors (specifically, patient’s and practitioner’s beliefs/characteristics; patient-practitioner relationships; the therapeutic setting/environment; and treatment characteristics) could be important, but there is limited evidence regarding their influence. This research aims to review the impact of interventions modifying contextual factors during conservative care on patient’s pain and physical functioning.

**Databases and data treatment:**

Four electronic databases (Medline, CINAHL, PsycINFO and AMED) were searched from 2009 until 15th February 2022, using tailored search strategies, and resulted in 3476 unique citations. After initial screening, 170 full-text records were potentially eligible and assessed against the inclusion–exclusion criteria. Thereafter, studies were assessed for methodological quality using a modified Downs and Black scale, data extracted, and synthesised using a narrative approach.

**Results:**

Twenty-one primary studies (*N* = 3075 participants), were included in this review. Eight studies reported significant improvements in pain intensity, and seven in physical functioning, in favour of the contextual factor intervention(s). Notable contextual factors included: addressing maladaptive illness beliefs; verbal suggestions to influence symptom change expectations; visual or physical cues to suggest pain-relieving treatment properties; and positive communication such as empathy to enhance the therapeutic alliance.

**Conclusion:**

This review identified influential contextual factors which may augment conservative chronic low back pain care. The heterogeneity of interventions suggests modifying more than one contextual factor may be more impactful on patients’ clinical outcomes, although these findings require judicious interpretation.

**Supplementary Information:**

The online version contains supplementary material available at 10.1186/s12998-022-00430-8.

## Introduction

Musculoskeletal (MSK) conditions are the second largest contributor to disability [1], with low back pain (LBP) being the single leading cause [2]. LBP is typified by pain and reduced physical functioning, often affecting mental health, and increasing risks for co-morbidities and all-cause mortality [3]. Chronic LBP (cLBP) frequently occurs in the absence of a known pathoanatomical cause (non-specific) and persists for 12 or more weeks [4]. Identified risk factors include lifting activities, smoking, obesity, and depressive symptoms, but these only increase the odds of developing cLBP by a modest amount [4]. Indirect LBP costs (e.g., carer-burden, decreased workforce participation) may exceed the direct costs [4] representing a threat to lifetime productivity and well-being [5].

Clinical guidelines recommend conservative treatments, specifically biopsychosocial approaches initially focusing on non-pharmacological treatment, [5], including exercise, massage, cognitive behavioural, and manual therapies [6] alongside comorbidity management, such a low mood, depression, or anxiety [4]. Systematic reviews support the use of non-steroidal anti-inflammatory drugs (NSAID) [7] and opioids [8] for cLBP, however, both have inherent long-term usage risks (e.g., opioid dependence; NSAID induced renal impairment). Moreover, when comparing the effectiveness of NSAIDS to placebos in studies with low risk of bias, the effect sizes were small [7]. Overemphasising biomedical or pharmacological care can result in poor health outcomes or iatrogenic consequences [3], with limited increased efficacy over conservative approaches [9]. Using ineffective, wasteful (e.g., overuse of imaging) or potentially deleterious practices exacerbates unsustainable healthcare expenditure, widening social and health inequalities [3, 10].

Beyond spontaneous or natural recovery, recent evidence suggests a considerable fraction of analgesic responses in treatments for MSK pain may be attributable to contextual factors (CFs) [11]. CFs are multidimensional (physical, social, and psychological) aspects of the clinical encounter capable of producing or inducing positive (placebo) or negative (nocebo) biological effects [12, 13]. Placebo mediated analgesia is a reduction in pain arising from features of the treatment context [12, 13] and involves defined endogenous neural pathways (e.g., dorsolateral prefrontal cortex, anterior cingulate cortex, periaqueductal grey and the dorsal horn of the spine), along with associated neurotransmitters (e.g., endogenous opioid, the endocannabinoid, and the dopaminergic systems), intrinsically linked to regions underlying conscious judgement of meaning [14–16]. Accordingly, pain modulation can potentially be induced by explicitly manipulating CFs [11, 12, 17] which Di Blasi and colleagues [18] characterised into five useful domains:

1. Patient’s beliefs and characteristics (*e.g., LBP history, gender, illness and treatment beliefs, expectations, or prior experiences*);
2. Practitioner’s beliefs and characteristics (*e.g., professional reputation, attire, empathy, professional training and prior experiences, and beliefs,*);
3. Patient-practitioner relationship (e*.g., therapeutic alliance, trust, verbal or non-verbal communication, reassurance*);
4. Therapeutic setting/environment (*e.g., setting, layout, décor, interior design*); and
5. Treatment characteristics (*e.g., continuity of care, labelling, visual cues, sham/dummy treatment, variations in touch or stimulus conditions*).

Although symptom improvement is a common treatment objective, other factors, such as the practitioner’s communication style (e.g., demonstrating genuine empathy), time-constraints (e.g., willingness and/or ability to listen), beliefs or treatment expectations, prior training, environmental conditions (e.g., interior design, environment, setting etc.) are likely to influence patients’ outcomes. Furthermore, there is a growing body of literature supporting explicit induction of placebo analgesia, as a clinically beneficial approach [11, 12, 16, 19], with outcomes similar in magnitude to treatment effects [20]. However, it remains unclear which elements of the therapeutic encounter are impactful on patient’s clinical outcomes.

Accordingly, a promising adjunct to care may involve overtly manipulating CFs to enhance treatment efficacy [12, 21] but there is limited evidence examining the influence of explicit manipulation of CFs on cLBP [11]. This systematic review therefore aims to examine interventions which potentially modify known CFs during conservative cLBP care (specifically, non-pharmacological, non-surgical, and non-invasive approaches) to investigate their impact on patients' pain intensity and physical functioning outcomes. Delineating the influence and role of CFs in usual care rehabilitation settings may assist in identifying which of these CFs demonstrates potential clinical utility and ethical approaches to rehabilitation.

## Materials and methods

The updated Preferred Reporting Items for Systematic Reviews and Meta-analyses (PRISMA; [22]) checklist was adhered to and the protocol was registered on PROSPERO (CRD42019145157).

### Eligibility criteria

Table 1 presents a summary of the eligibility criteria. Only studies available in full-text were included to ensure adequate appraisal and review. The following limits were applied: human studies published in English between 2009 and 2022. The justification for this period was twofold. The primary rationale was to ensure uniformity in conservative care approaches across potentially eligible studies. The National Institute for Health and Care Excellence (NICE) guidelines for non-invasive treatments for LBP [23] guided this decision. The secondary justification is conceptual: there is a lack of definitional consensus, coupled with an array of interchangeable concepts, which are evolving in tandem with emergent knowledge, but no unified theory [24]; consequently, historical interpretations and associated research may not be wholly aligned with the CF framework.

**Table 1:** Summary of inclusion–exclusion criteria

	Inclusion	Exclusion
Population/Illness	Adult (≥ 18 years) with chronic low back pain (≥ 12 weeks)	Non-human subjects; human objects (e.g., tissues, fMRI, MRI, CT etc.), children and adolescents (< 18 years old); fictitious/actor patients, patients with acute, sub-acute or mixed samples of low back pain
Treatment Setting	Universally accepted/clinically recognised forms of conservative care (i.e., non-pharmacological and non-invasive) occurring in a clinical setting (in-patient or out-patient), primary or secondary healthcare (private or public) or where it was expressly articulated that the site involves regular delivery of therapeutic care (e.g., University clinic, community care centre)	Excluded if treatment related to palliative care, emergency medicine or experimental laboratory environments
Intervention	Conservative care approaches/strategies which potentially alter clinical outcomes through the explicit modulation or measurement of at least one of the five contextual factors relating to the health encounter	Excluding pharmacological or surgical interventions; acupuncture, injections, or invasive procedures; neurological experiments or imaging; interventions targeting adherence to analgesic medication, diet modification/nutritional supplements; interventions involving alternative medicine; online, app-based or telehealth interventions
Comparators	No treatment or intervention (e.g., waiting-list control or natural history group), no control group (i.e., uncontrolled study), standard/usual care, or neutral, negative, or an experimentally dosed and/or opposite or contextually comparative condition	Two-armed trial or two-group design whereby the description indicates a standard placebo-controlled trial (where the comparison group involved a “sham” condition perceived to be an “inert” placebo)
Outcomes	Validated pain or physical functioning/disability measures (e.g., used during routine clinical care)	Non-validated pain or physical functioning/disability measures or sub-scales
Study Design	Randomised Controlled Trials (RCTs); quasi-experimental designs, or prospective longitudinal studies	Retrospective/secondary analyses, qualitative studies, cross-sectional designs, n-of-1 trial; conference abstracts, dissertations, and trial protocols

To further clarify, an eligible intervention involved strategies designed to change or potentially modify one or more known contextual factor(s) of the health encounter/clinical consultation or experimental condition. This was guided by the review teams’ understanding of the theoretical mechanisms important to generating placebo analgesia such as classical conditioning, expectancy theory, social or experiential learning, predictive coding, and the Bayesian brain (see [25]). For instance, strategies involving manipulating patients’ or practitioners’ expectations, beliefs, perceptions, learned associations, mindsets, aspects of their interpersonal communication, appearance/clothing, aspects of the patient-practitioner relationship, the environment (e.g., setting, décor, place, waiting time), varying packaging, patient information leaflets (e.g., drug effects, side effects, adverse events), sham devices or procedures, labels, differential pricing, warning labelling, and so forth to influence patient outcomes either before, during, after or throughout the treatment duration. Studies of both positive and negative interventions, namely, those specifically designed to induce placebo effects or nocebo effects were eligible. It is possible that there are psychological interventions that may not (currently) be known to induce placebo analgesia, such as general patient education. Such interventions were eligible if it was clear that it intended to alter the patient’s expectations (e.g., influence pain perception) as this is consistent with theories of placebo mediated analgesia which assume a prediction is made (whether conscious or not) about a future health state. Such anticipatory processes are effectively based on the interpretation of both internal and external factors (which are purported to be psychological meaningful) and capable of triggering an associated neurobiological response [14].

Accordingly, eligible interventions could be simple or complex; and involve an extensive array of CFs, placebo effects, or situational elements intended to influence the design of the health encounter or the treatment of cLBP. Multimodal interventions modifying one or more CF(s) combined with usual care were included if the control group involved a well-controlled comparison condition as defined by Howick and colleagues [26]. In an experimental condition, it could involve covert (hidden design), or overt (open design) tactics expected to induce a placebo effect, or prevent a nocebo effect, such as parallel group design (e.g., three-arm trial), response conditioning design, open versus hidden design, or pharmacological conditioning designs (see [14]). Uncontrolled studies reporting on clinical outcomes which involved modification of a CF(s) (e.g., the new component was introduced as part of routine care) as well as prospective longitudinal studies where a CF(s) was pre-existing (e.g., association between outcomes after increasing consultation times or the pre-existing relationship between the patient and their healthcare provider) were also eligible. There was no limit on the length of the intervention, such as the number of sessions or time/period, provided the intervention occurred in a setting or site involving the regular delivery of therapeutic care for cLBP. Individual or group-based interventions were potentially eligible. Online, or app-based interventions were excluded because these may not be aligned with the conceptual framework of CFs since there are negligible patient-practitioner interactions and it is not a traditional clinical setting.

### Search procedure

#### Information sources

Studies were identified using the following databases: Medline (via ProQuest); Cumulative Index to Nursing and Allied Health Literature (CINAHL via EBSCOhost); PsycINFO (via ProQuest); and Allied and Complementary Medicine (AMED via Ovid) from 2009 until the search date (15th February 2022). Additionally, named author searches (via Google scholar) and manual searches of reference lists of provisionally eligible primary studies, and the Journal of Interdisciplinary Placebo Studies (JIPS) database were conducted to identify studies potentially undetected through electronic searching.

#### Search

Search strategies (see Additional file 1: Search Strategy Methods S1–S4) were tailored per database using suitable Boolean operators, phrase searching, and Medical Subject Headings (exploded where appropriate) using key concepts and their alternatives (see Table 2). Key concepts included: (1) chronic low back pain; (2) placebo effects/contextual factors; (3) healthcare professionals and patient relationships/interactions; as well as (4) healthcare professionals and patient expectations/beliefs. Searches were limited to title and abstract to ensure standardisation across databases, and then screened for eligibility once duplicates were removed.

**Table 2:** Examples of search terms for key concepts

Key concepts	Search terms
Chronic low back pain	“back pain”, “low back pain”, LBP, “chronic low back pain”, cLBP, “non?specific low back pain”, “non?specific back pain”, “lumbar pain”
Placebo effects/Contextual Factors	(placebo ADJ (effect* OR response* OR analgesi*)), (nocebo ADJ (effect* OR response*), (context* ADJ (factor* OR effect* OR response*)), (common ADJ (factor* OR effect*)), (non?specific ADJ (effect* OR factor*))
Healthcare professionals and patient relationships/interactions	alliance*, (patient ADJ (relation* OR interact*)), (empath* OR warm* OR compassion* OR kind* OR friendl*), rapport, “non?verbal communication*”, “verbal communication*”, “health communication*”, "initial consultation", "professional-patient relation*","physician-patient relation*"
Healthcare professionals and patient expectations/beliefs	(patient* ADJ (expect* OR belief* OR attitude*)), (practitioner* ADJ (expect* OR belief* OR attitude*)), (positive ADJ (expect* OR suggest*)), (negative ADJ (expect* OR suggest*)), illness ADJ (perception* OR belief*)

### Study selection

#### Screening

Initially citations were screened by title and abstract based on the eligibility criteria. A conservative approach was employed—in cases of uncertainty, the record was retained for full-text screening. Thereafter, full-text papers were assessed using a standardised, pre-piloted screening proforma, along with documenting reasons for exclusion and identifying studies reporting on the same dataset. Both screening and selection stages were carried out by the primary reviewer (BS). In addition, the entire review team also cross-checked a proportion (*n* = 50; 29.4%) of potentially eligible full-text articles. Any discrepancies in opinion were resolved through discussion and a final adjudication was made using a consensus-based approach.

#### Quality appraisal

Eligible studies were assessed for methodological quality using a modified Downs and Black scale consisting of 27 items [27]. This tool was selected as it is appropriate for assessing both randomised and non-randomised studies, the reliability is reportedly high (internal consistency – Kuder–Richardson-20: 0.89; test–retest reliability: *r* = 0.88), [27] and has previously been used in other systematic reviews [28–30]. This tool has five sub-sections, namely, quality of reporting (ten items); external validity (three items); bias (seven items); selection bias/confounding (six items); and statistical power (one item). The scoring of statistical power (item 27) was amended from five points to one (following [29, 31]), altering the total score to 28. Following O’Connor and colleagues [31], each study was graded “Excellent” (24–28 points), “Good” (19–23 points), “Fair” (14–18 points) or “Poor” (< 14 points). Owing to the inherent design of observational and single-group experiments, inapplicable questions were removed (e.g., random assignment, group allocation and concealment) and scoring adjusted accordingly.

#### Data extraction

The primary reviewer (BS) extracted data using a proforma, adapted from the Template of Intervention Description and Replication (TIDieR) [32] and the Cochrane Effective Practice and Organisation of Care Review Group (EPOC) [33] data collection checklist. The following data were extracted: -

i) *Study identification features*: author(s), year of publication, title, country of origin, setting, theoretical model/basis, and aim(s).
ii) *Study features*: study design, inclusion and exclusion criteria, recruitment method, data collection method, length of follow-up, (specifically timing of measures), method of random assignment, main statistical analysis.
iii) *Sample characteristics*: intervention group (*n*), comparison group(s) (*n*), total sample size (*n*), description of the population (specifically gender proportions, mean/median age, mean/median duration of cLBP), mean/median baseline pain intensity and/or physical functioning scores and standard deviations.
iv) *Intervention description*: type of contextual factor(s), intervention components, delivery format, treatment frequency, treatment duration, number of session(s), length of treatment session(s), administering practitioner(s), type of comparison/control group(s), description of comparison/control conditions.
v) *Main Results*: measure(s) of pain intensity and/or physical functioning outcomes, post-treatment, and follow-up (if applicable) mean pain intensity and/or physical functioning scores, standard deviations, p-values, effect sizes, and main findings relevant to the review aim(s).

#### Data synthesis

A narrative synthesis was applied to the extracted data guided by the Economic and Social Research Council (ESRC) Methods Programme framework (see [34]). The synthesis process was iterative and nonsequential, rather than linear, thereby facilitating general inferences to be delineated regarding CFs and their impact on cLBP patients’ pain intensity and physical functioning outcomes. Both within and between group data were tabulated to identify influential CFs in relation to these two main outcomes. Not all of the included studies investigated both within and between groups differences. The absence of such data is not a result of reporting bias but rather the heterogeneity of research designs and corresponding study aims included in this review.

## Results

### Search results

**Fig. 1:**
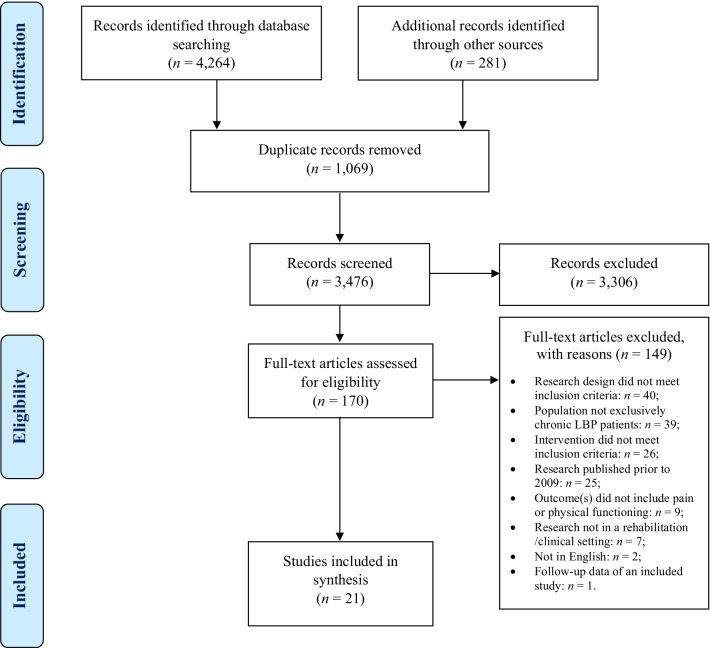
Modified PRISMA flow chart diagram. This figure shows the citations through the selection process. 4545 records were identified, and after removing 1069 duplicates, the remaining 3476 titles and abstracts were screened. Out of these, 170 full-text articles were assessed for eligibility, and 21 were included in the final selection and data synthesis

The electronic and manual searches resulted in 3476 unique citations, of which, 21 met the eligibility criteria. Using a modified PRISMA flow chart, Fig. 1 illustrates how these studies were selected.

### Quality assessment

The overall risk of bias across studies was relatively low; 13 were graded as ‘Excellent’ [35–41, 47, 50, 52–55], seven as ‘Good', [42–46, 48, 51] and only one as ‘Fair’ [49]. ‘Good’ ratings were generally on the higher end of the scoring spectrum but the common distinction from an ‘Excellent’ grading related to the external validity sub-scale (items 11 and 13), and/or statistical power (item 27) where 11 (52.4%), nine (42.9%), and 11 studies (52.4%) were scored negatively respectively (see Table 3 summary).

**Table 3:** Quality assessment summary clustered by research design

Reference (year)	Quality of reporting (*10 items*) Range: 0–11 points	External validity (*3 items*) Range: 0–3 points	Internal validity (*7 items*) Range: 0–7 points	Selection bias/confounding (*6 items*) Range: 0–6 points	Statistical power (*1 modified item*) Range: 0–1 points	Total score (27 items) Range: 0–28 points	Overall grading Excellent (24–28) Good (19–23) Fair (14–18) Poor (< 14)
**Randomised controlled trials** (RCTs)							
[35] (2011)	11	2	7	6	0	26 (92.9%)	Excellent
[36] (2013)	10	3	7	5	0	25 (89.3%)	Excellent
[37] (2014)	11	0	7	6	1	25 (89.3%)	Excellent
[38] (2022)	11	2	6	5	1	25 (89.3%)	Excellent
[39] (2019)	11	1	6	6	1	25 (89.3%)	Excellent
[40] (2016)	10	2	6	6	0	24 (85.7%)	Excellent
[41] (2017)	10	3	7	4	0	24 (85.7%)	Excellent
[42] (2010)	10	1	6	6	0	23 (82.1%)	Good
[43] (2020)	10	2	5	5	1	23 (82.1%)	Good
[44] (2021)	10	2	4	6	1	23 (82.1%)	Good
[45] (2017)	10	0	6	5	1	22 (78.6%)	Good
[46] (2019)	9	2	6	3	0	20 (71.4%)	Good

Reference (year)	Quality of reporting (*10 items*) Range: 0–11 points	External validity (*3 items*) Range: 0–3 points	Internal validity (*7 items*) Range: 0–7 points	Selection bias/confounding (*4 items*) Range: 0–4 points	Statistical power (*1 modified item*) Range: 0–1 points	Total score (*25 items*) Range: 0–26 points	Overall grading Excellent (22–26) Good (18–21) Fair (13–17) Poor (< 13)
**Controlled clinical trials** (CCT; non-randomised)^**a**^							
[47] (2017)	10	2	6	4	1	23 (88.5%)	Excellent
[48] (2012)	10	1	5	3	0	19 (73.1%)	Good
[49] (2018)	7	1	5	3	1	17 (65.4%)	Fair

Reference (year)	Quality of reporting (*10 items*) Range: 0–11 points	External validity (*3 items*) Range: 0–3 points	Internal validity (*5 items*) Range: 0–5 points	Selection bias/confounding (*2 items*) Range: 0–2 points	Statistical power (*1 modified item*) Range: 0–1 points	Total score (*21 items*) Range: 0–22 points	Overall grading Excellent (19–22) Good (16–18) Fair (11–15) Poor (< 11)
**Quasi-experimental** (uncontrolled)^**b**^							
[50] (2015)	9	3	5	2	0	19 (86.4%)	Excellent
[51] (2017)	10	1	5	2	0	18 (81.8%)	Good

Reference (year)	Quality of reporting (*9 items*) Range: 0–10 points	External validity (*3 items*) Range: 0–3 points	Internal validity (*5 items*) Range: 0–5 points	Selection bias/confounding (*3 items*) Range: 0–3 points	Statistical power (*1 modified item*) Range: 0–1 points	Total score (*21 items*) Range: 0–22 points	Overall grading Excellent (19–22) Good (16–18) Fair (11–15) Poor (< 11)
**Observational Cohort** (uncontrolled)^**c**^							
[52] (2013)	10	3	5	3	0	21 (95.5%)	Excellent
[53] (2013)	10	2	5	3	0	20 (90.9%)	Excellent
[54] (2011)	10	2	4	2	1	19 (86.4%)	Excellent
[55] (2019)	8	2	5	3	1	19 (86.4%)	Excellent

The following inapplicable items were not included in the quality assessment for this study design:

^**a**^Selection bias sub-scale: -Q23. Were study subjects randomised to intervention groups?; Q24. Was the randomised intervention assignment concealed from both patients and health care staff until recruitment was complete and irrevocable?

^**b**^Internal validity sub-scale:—Q14. Was an attempt made to blind study subjects to the intervention they have received?; Q15. Was an attempt made to blind those measuring the main outcomes of the intervention? Selection bias sub-scale:—Q22. Were study subjects in different intervention groups (trials and cohort studies) or were the cases and controls (case–control studies) recruited over the same period of time?; Q23. Were study subjects randomised to intervention groups?; Q24: Was the randomised intervention assignment concealed from both patients and health care staff until recruitment was complete and irrevocable?; Q25. Was there adequate adjustment for confounding in the analyses from which the main findings were drawn?

^**c**^Reporting sub-scale: -Q8. Have all important adverse events that may be a consequence of the intervention been reported?; Internal validity sub-scale:—Q14. Was an attempt made to blind study subjects to the intervention they have received?; Q15. Was an attempt made to blind those measuring the main outcomes of the intervention?; Selection bias sub-scale:—Q22. Were study subjects in different intervention groups (trials and cohort studies) or were the cases and controls (case–control studies) recruited over the same period of time?; Q23. Were study subjects randomised to intervention groups?; Q24: Was the randomised intervention assignment concealed from both patients and health care staff until recruitment was complete and irrevocable?

Of 11 studies with a zero rating for statistical power (item 27), five were underpowered [36, 40, 42, 46, 48], whilst it was unclear/undetermined for the remaining six [35, 41, 50–53]. By implication, the between-group results may be understated, since four of 15 comparative studies (3 RCTs and 1 CCT) [35, 41, 42, 48] reporting non-significant differences between groups were potentially underpowered. If corresponding confidence intervals were consistently reported, it would facilitate a clearer adjudication of these results.

Global estimates for LBP were extrapolated to create a rudimentary set of criteria to assess external validity (item 11) and uniformly applied to each study’s sample. LBP is typically more common in females, but these differences appear to diminish once chronicity is accounted for [56] whilst age-related LBP prevalence is generally negatively skewed and reported to be highest between 40 to 69 years [4] whilst global LBP prevalence reportedly peaks around 80 years old [57]. Accordingly, nine studies [36, 38, 41, 44, 46, 47, 50, 52, 54] scored ‘1’ for satisfying both conditions: (i) the proportion of females is higher but less than 60% overall; and (ii) the mean/median age falls within the range of 40.00 to 63.5 years (but 10 and 17 studies satisfied one condition respectively – see Additional file 1: Item 11 scoring grid Results S1). Since comorbid and/or confounding conditions (e.g., age restrictions, pregnancy, neurological, rheumatological, cancer, fractures, recent surgery) were generally excluded, these samples are fairly homogenous since their inclusion–exclusion criteria were comparable, but older patients were typically excluded.

Similarly, item 13, pertains to the representativeness of the staff, facilities, intervention and setting the majority of patients would typically have access to or receive. Studies scoring ‘1’ should demonstrate that the intervention was representative of that in use in the source population. Given the geographic variability between studies, what is considered typical treatment for cLBP differs across settings and regions. Although not universally applicable, the NICE guideline [23] for non-invasive LBP treatments guided the assessment. Studies receiving a zero rating involved the following: three employed experimental techniques (namely classical conditioning, and sham versus *verum* interferential current therapy (IFC)) [37, 45, 46]; two offered a single educational pain biology session (not specifically encouraging self-management behaviours) [42, 51]; and four used cognitive behavioural approaches but were not combined with exercise and/or manual therapies [38, 44, 48, 54].

### Study characteristics

Twenty-one studies (*N* = 3075 participants) with a wide range of research designs were included in the review; specifically, 12 randomised clinical trials (RCTs; *n* = 1064 [35–40, 42–46]; *n* = 255 cluster-randomised [41]), three non-randomised, controlled clinical trials (CCTs; *n* = 460) [47–49], four observational cohort studies (*n* = 1220) [52–55], one case series (*n* = 50) [51], and one interrupted time series (*n* = 26) [50]. RCT sample sizes ranged from 38 (pilot [42]) to 222 (3-armed trial [44]) patients. Across the remaining studies, sample sizes ranged from 26 (interrupted time series [50]) to 688 participants (prospective cohort; [52]). All samples consisted of adult patients with cLBP; mean ages ranged from 30 to 66.8 years, whilst the mean duration of LBP varied considerably (ranging from 3–12 months up to 18.5 years). There were higher ratios of female patients in all studies except one [49], whilst the cumulative gender proportions were skewed towards females (59.1% female; *n* = 1761; 40.9% male; *n* = 1219; (95 missing cases)). The studies were predominantly clustered in the Northern hemisphere but geographically diverse, originating from twelve countries. Fourteen settings involved single-centre treatment/rehabilitation clinics, whilst seven involved multiple-centres. Only one study explicitly indicated that the intervention took place in a private healthcare setting [36], and another involved a combination of both in-patient and out-patient orthopaedic rehabilitation centres [52].

A variety of outcome measures were reported; pain intensity was most commonly measured using a Numeric Rating Scale ((NRS);16 studies) whilst four studies utilised a Visual Analogue Scale (VAS), and one did not include pain severity as an outcome [54]. Eight studies employed the Roland-Morris Disability Questionnaire (RMDQ), eight the Oswestry Disability Index (ODI), and one did not measure physical functioning [37]. The remaining studies utilised the following measures: Patient Specific Functional Scale (PSFS) [39, 44, 54], the Hannover Activities of Daily Living Questionnaire (ADL) accompanied by the specified activities [45, 46], a lumbar flexion test operationalised as the change in distance between the fingertips to the floor [51], and a Timed-Up-and-Go (TUG, measured in seconds) [43]. Three studies used more than one measure of physical functioning, namely, the ODI and PSFS [39, 44] and the RMDQ and TUG [43]. Refer to Additional file 1: Table S1 for a summary of the key characteristics of the included studies.

### Overall influence of contextual factors

Across the 21 studies, patient’s beliefs were the most commonly manipulated (16 studies) [35, 36, 38–48, 50, 51, 54] or measured CF (1 study) [55] followed by the patient-practitioner relationship (nine studies) [35, 37, 41, 42, 44, 47, 49, 52, 53], and the treatment characteristics (seven studies) [35, 37, 39, 40, 43, 45, 46] whilst only one modified the treatment context [49]. Nine modified (or measured) one CF only [36, 38, 48, 50–55] while 12 modified two or more CFs [35, 37, 39–47, 49]. None of the included studies examined the influence of practitioner beliefs and characteristics. Assessing both between-group differences and within-group differences delineates the overall impact of CFs on patient outcomes.

#### Within-group differences: pain intensity and physical functioning

Considering only the CF-intervention arm(s) across the 21 included studies, nine demonstrated statistically significant differences in pain intensity within-groups [35, 36, 45, 46, 48–52], whilst one did not measure it [54]. The overall trend was a reduction in pain intensity over time, as another nine studies [37–42, 44, 47] also demonstrated improvements, although relevant test-statistics and/or corresponding *p*-*values* were not reported. Both quasi-experimental studies reported 54% and 42% of patients achieved a minimal clinically important difference (MCID) in pain intensity after receiving treatment modifying CFs [50, 51]. Two studies reported clinically meaningful improvements [37, 38]. In the RCT using either active or sham inferential current therapy (IFC), the two enhanced therapeutic alliance groups both reported 77.4% and 54.5% improvements in pain intensity respectively [37]. Similarly, in the Pain Reprocessing Therapy (PRT) trial [38], 78% of patients experienced more than a 30% reduction in pain intensity at post-treatment and 70% at 1-year follow-up. In the Portuguese open-label placebo (OLP) trial [40], the CF-manipulation arm experienced a 28% reduction in pain intensity which falls shy of a clinically meaningful improvement (30% reduction). Two observational cohorts reported significant relationships between therapeutic alliance and pain [53] and patient’s competence perceptions and pain [55] respectively. However, the Japanese OLP trial reported no statistically significant improvements from baseline, but 45.8% of patients experienced ≥ 2-unit change in pain intensity at 12-weeks follow-up [43].

Correspondingly, 20 studies reported within-group differences in respect of physical functioning outcomes; of these, ten demonstrated statistically significant improvements from baseline [35, 36, 43, 45, 46, 48–52] whilst one did not include disability as an outcome [37]. Seven studies reported the mean differences but did not include relevant test-statistics nor *p-values* [38–42, 44, 47], but the general trend was an overall improvement in physical functioning from baseline. For example, both quasi-experimental studies reported 62.5% and 36% of patients achieved a MCID after treatment modifying CFs [50, 51], and a larger improvement was reported in the CF-manipulation arm compared to the control arm in a non-randomised CCT [47]. The Portuguese OLP group experienced a 29% improvement in physical functioning compared to 0% (no change) in the treatment as usual arm [40], whilst the Japanese OLP trial reported significant changes in RMDQ scores but not TUG times from baseline [43]. Additionally, three observational cohorts reported significant relationships between therapeutic alliance and physical functioning [53], patient’s rational problem-solving skills and physical functioning [54] as well as patient’s competence perceptions and physical functioning [55]. Overall, these within-group improvements suggest that interventions involving CFs are influencing pain intensity and physical functioning outcomes in patients with cLBP over time. Refer to Additional file 1: Table S2 for a summary of within-group changes in outcomes from baseline clustered by research design.

#### Between-group differences: pain and physical functioning

Fifteen studies involved two or more treatment arms; of these, eight (of 12) RCTs demonstrated statistically significant differences in pain intensity between groups in favour of the CF-manipulation [36–40, 42, 45, 46] as illustrated in Table 4. One three-armed trial only demonstrated significant differences at 12-months follow-up [44] between each arm receiving an educational intervention compared to the group receiving no education, but there were no differences between the two groups receiving the educational intervention (one with an emphasis on developing the therapeutic alliance). Of these eight RCTs, six modified more than one CF, and four [37–39, 45] were adequately powered (80%; α = 0.05) to detect changes in pain intensity. The remaining six failed to demonstrate statistically significant differences between groups regarding pain intensity [35, 41, 43, 47–49]. Of these, three were RCTs [35, 41, 43], three were non-randomised CCTs [47–49] and three of these studies were adequately powered [43, 47, 49]. However, at 12-months follow-up, one CCT reported the CF-manipulation arm had significantly lower ‘*worst pain*’ ratings, but not significantly lower ‘*average pain*’ ratings compared to conventional physical therapy [47]. In one RCT, a significant increase in pain intensity (potential nocebo effect) was reported in one of the four treatment conditions – *open-label placebo instruction without conditioning arm* [45].Regarding physical functioning outcomes, seven of the fourteen studies demonstrated statistically significant differences between groups in favour of the CF-intervention [36, 38–40, 44–46], all of which were RCTs, and five modified more than one CF. Of these, four studies were adequately powered [38, 39, 44, 45]. At 12-months follow-up, one CCT reported the CF-manipulation arm had significantly improved physical functioning compared to conventional physical therapy but there was no between-group difference at post-treatment [47]. The remaining six studies failed to demonstrate between-group differences in physical functioning [35, 41–43, 48, 49], but only two were adequately powered [43, 49]. Notably, one RCT observed that sex moderated the intervention’s effect, where women in the CF-intervention arm improved 4.94 RMDQ points compared to the usual care physiotherapy group [41].

**Table 4:** Between-group comparisons in outcomes clustered by research design

Ref no. (Year) Design [Grading]	CF-intervention	Comparison condition(s)	Between-group difference Mean (*SD*) Pain Intensity	Intervention’s influence: pain intensity	Between-group difference Mean (*SD*) Physical functioning	Intervention’s influence: Physical functioning
[35] (2011) RCT [Excellent]	Motivational Enhancement Treatment (**MET**) + PT: (*n* = 38) included proxy efficacy, treatment expectancy, therapeutic alliance, and empathy, combined with physical therapy	**PT** (*n* = 38): 30-min physical therapy (PT) sessions for 8 weeks, including 15 min of interferential therapy and a tailored back exercise programme. Dummy MET included general communication (non-counselling) skills	**MET + PT** Post: *M* = 3.3 (± 2.1) 1-month*: M* = 3.1 (± 2.1) **PT only:** Post: *M* = 3.6 (± 2.4) 1-month: *M* = 3.9 (± 2.5)	**MET + PT ~ PT** (*p* = .50) 95% C.I. [− 1.09 to 0.54] MET + PT larger reduction in pain intensity than PT-alone but *non-significant*	**MET + PT** *Post: M* = 6.3 (± 4.8) 1-month: *M* = 5.6 (± 4.5) **PT only** *M* = 7.2 (± 5.6) 1-month: *M* = 7.6 (± 6.4)	**MET + PT ~ PT** (*p* = .424) 95% C.I. [− 2.83 to 1.44] MET + PT larger reduction in physical disability than PT-alone but *non-significant*
[36] (2013) RCT [Excellent]	Cognitive Functional Therapy **(CFT)** (*n* = 51): focuses on reframing back pain in a person-centred manner along with altering maladaptive/unhelpful behaviours to normalise movement	**MT-EX** (*n* = 43): consisted of manual therapy and exercise which included joint mobilisation or manipulation applied to the spine or pelvis; most patients (82.5%) were given exercises or a home exercise programme	**CFT** Post: *M* = 1.7 (± 1.7) 1-year: *M* = 2.3 (± 2.0) **MT-EX** Post: *M* = 3.8 (± 1.9) 1-year: *M* = 3.8 (± 2.1)	**CFT > MT-EX** Post: **(*p* < .001)** *M*_*Diff*_ = − 2.1 95% C.I. [− 2.7 to − 1.4] 1-year**: (*p* < .001)** *M*_*Diff*_ = − 1.3 95% C.I. [− 2.1 to − 0.5] *Effect size unreported*	**CFT** Post: *M* = 7.6 (± 6.7) 1-year: *M* = 9.9 (± 9.8) **MT-EX** Post: *M* = 18.5 (± 8.1) 1-year:*M* = 19.7 (± 11.7)	**CFT > MT-EX** Post: **(*p* < .001)** *M*_*Diff*_ = − 9.7 95% C.I. [− 12.7 to − 6.7] 1-year: **(*p* < .001)** *M*_*Diff*_ = − 8.2 95% C.I. [− 12.6 to − 3.8] *Effect size unreported*
[37] (2014) RCT (2 × 2) [Excellent]	Enhanced versus limited therapeutic alliance (**TA**) following active or sham interferential current therapy (**IFC**)	Variation of CFs: Enhanced TA ( **E** ): **AE**: Active IFC (*n* = 29) **SE**: Sham IFC (*n* = 29) Limited TA ( **L** ): **AL**: Active IFC (*n* = 30) **SL**: Sham IFC (*n* = 29)	Significant differences between the SL and the AL, AE & SE groups Compared to **SL** (Sham IFC/Limited TA) mean differences were: **AE**: *M*Δ = 2.3 **SE**: *M*Δ = 1.19 **AL**: *M*Δ = 0.8	**(** ***p*** **< .01)** Dose response AE > SL: *d* = 2.51 **Enhanced > Limited TA** SE > SL: *d* = 1.73 AE > AL: *d* = 1.36 **Active > Sham IFC** AE > SE: *d* = 1.0 AL > SL: *d* = 0.89	*Not applicable*	*Not applicable*
[38] (2022) RCT [Excellent]	**PRT** (*n* = 50): Pain Reprocessing Therapy (PRT) aims to shift patients’ beliefs about the causes and threat value of pain	**TAU** (*n* = 50): Treatment as usual; Participants were given no additional treatment and agreed to continue their ongoing care as usual and not start new treatments before the post-treatment assessment	**PRT** Post: *M* = 1.18 (± 1.24) 1-year: *M* = 1.51 (± 1.59) **TAU** Post: *M* = 3.13 (± 1.45) 1-year: *M* = 3.0 (± 1.77)	**PRT > TAU** Post: **(*p* < .001)** *g* (SE) = − 1.75 (0.24) 1-year: **(*p* < .001)** *g* (SE) = − 1.05 (0.24)	**PRT** Post: *M* = 10.14 (± 10.6) 1-year: *M* = 11.16 (± 13.1) **TAU** Post: *M* = 20.68 (± 10.7) 1-year: *M* = 18.78 (± 12.6)	**PRT > TAU** Post: **(*p* < .001)** *g* (SE) = − 1.70 (0.26) 1-year: **(*p* < .001)** *g* (SE) = − 0.83 (0.24)
[39] (2019) RCT [Excellent]	**OLP** (*n* = 63): Open-label placebo pills, social learning with **TAU**	**TAU** (*n* = 59): Treatment as usual (TAU) patients received no intervention (*no further description provided*)	**OLP + TAU**: Post: *M*Δ = –0.62 (± SE = 0.23) **TAU**: Post: *M*Δ = 0.11 (± SE = 0.17)	**OLP + TAU > TAU** Post: **(*p* = .001)** *d* = –0.44	**OLP + TAU**: Post: *M*Δ = 23.21 (SE ± 1.59) **TAU**: Post: *M*Δ = 0.65 (± SE = 1.15)	**OLP + TAU > TAU** Post: **(*p* = .02)** *d* = –0.45
[40] (2016) RCT [Excellent]	**OLP** (*n* = 41): Open-label placebo pills, verbal suggestions, social learning with **TAU**	**TAU** (*n* = 42): Treatment as usual in an outpatient pain unit of a general public hospital (*no further description of treatment provided*)	**OLP + TAU**: *M*Δ = 1.49 (± 1.68) **TAU**: *M*Δ = 0.24 (± 1.61)	**OLP + TAU > TAU** Post: **(*p* < .001)** *g* = 0.76	**OLP + TAU**: *M*Δ = 2.86 (± 3.91) **TAU**: *M*Δ = 0.02 (± 3.73)	**OLP + TAU > TAU** Post: **(*p* < .001)** *g* = 0.74
[41] (2017) RCT (Cluster) [Excellent]	Communication Style and Exercise Compliance in Physiotherapy (**CONNECT**) (*n* = 108): Enhancing physiotherapists’ communication skills to alter unhelpful patient beliefs and improve motivation	**TAU** (*n* = 99): Treatment as usual; publicly funded physiotherapy with no restrictions on the number of sessions or the type of treatment the physiotherapist administered	**CONNECT** Post: *M*Δ = − 1.53 (± 2.71) 24 weeks: *M*Δ = − 1.53 (± 2.78) **TAU** Post: *M*Δ = − 1.31 (± 2.36) 24 weeks: *M*Δ = − 1.18 (± 3.19)	**CONNECT ~ TAU** (*p* = .75) *M*_*Diff*_ = − 0.10 95% C.I. [− 0.71 to 0.51] *d* = − 0.04	**CONNECT** Post: *M*Δ = − 3.48 (± 5.72) 24 weeks: *M*Δ = − 4.87 (± 5.86) **TAU** Post: *M*Δ = − 2.82 (± 5.77) 24 weeks: *M*Δ = − 4.09 (± 5.95)	**CONNECT** ^a^ **~ TAU** (*p* = .60) *M*_*Diff*_ = − 0.36 95% C.I. [− 1.68–0.96] *d* = − 0.08
[42] (2010) RCT [Good]	**ED** (*n* = 18): Pain biology education for the management of cLBP	Variation of CFs: **ED-EX (***n* = 20): Pain biology education plus six weekly exercise sessions (in a group format)	**ED** Post: *M*Δ = − 30.9 **ED-EX** Post: *M*Δ = − 4.2	**ED > ED-EX** (*p* = .025)	**ED** Post*: M*Δ = − 7.5 **ED-EX** Post: *M*Δ = − 3.8	**ED ~ ED-EX** (*p* = .127)
[43] (2020) RCT [Good]	**OLP** (*n* = 26): Open-label placebo pills, verbal suggestions, with TAU (advice, education, reassurance, self-management)	**TAU** (*n* = 26): Treatment as usual included advice to remain active, along with education and reassurance in addition to a psychological education self-management strategy to improve pain-related disabilities	**OLP + TAU**: Post: *M*Δ = –0.9 (± 1.8) 12-weeks: *M*Δ = –1.1 (± 1.9) **TAU**: Post: *M*Δ = –0.2 (± 1.8) 12-weeks: *M*Δ = –0.8 (± 1.9)	**OLP + TAU ~ TAU** Post: (*p* = .19) *d* = 0.38 12-weeks: (*p* = .18) *η*_*p*_^*2*^ = 0.04	**OLP + TAU**: RMDQ: *M*Δ = –2.2 (± 2 .9) TUG: *M*Δ = –0.7 (± 1.0) 12-weeks: RMDQ: *M*Δ = –3.3 (± 3.2) TUG: *M*Δ = –0.62 (± 1.5) **TAU**: RMDQ: *M*Δ = –1.4 (± 3.6) TUG: *M*Δ = –0.7 (± 1.5) 12-weeks: *RMDQ: M*Δ = –2.3 (± 3.2) TUG: *M*Δ = –1.1 (± 1.1)	**OLP + TAU ~ TAU** **RMDQ:** (*p* = .40) *d* = 0.24 **TUG:** (*p* = .98) *d* = 0.01 12-weeks **RMDQ**: (*p* = .37) *η*_*p*_^*2*^ = 0.02 **TUG:** (*p* = .28) *η*_*p*_^*2*^ = 0.03
[44] (2021) RCT [Good]	**ED + TA** (*n* = 74): Patient education (ED) relating to return to daily activities, advice on coping with pain, a clear explanation of signs and symptoms with an emphasis on increasing empathy and therapeutic alliance (TA)	Variation of CFs: **ED only** (*n* = 74): the same structured patient education sessions (ED) but with no emphasis on enhancing the patient-practitioner relationship **No ED** (*n* = 74): Patients received no-education and were advised not to seek treatment in the first month after randomisation	**ED + TA** ***vs*** **ED only** Post: *M*_*Diff*_ = 0.09 6-mo: *M*_*Diff*_ = 0.61 1-year: *M*_*Diff*_ = − 0.02 **ED + TA** ***vs*** **No ED** Post: *M*_*Diff*_ = 0.06 6-mo: *M*_*Diff*_ = − 0.05 1-year: ***M***_***Diff***_** = 1.40** **ED only *vs* No ED**: Post: *M*_*Diff*_ = 0.15 6-mo: *M*_*Diff*_ = 0.55 1-year: ***M***_***Diff***_** = 1.37**	**ED + TA ~ ED only** *ns:* (*p*-values unreported) **ED + TA > No ED** 1-year: **(*p* < .05)** Post & 6-months: *ns* **ED only > No ED** 1-year: **(*p* < .05)** Post & 6-months: *ns* *Effect sizes unreported*	*M*_*Diff*_ **PSFS/ODI** **ED + TA** ***vs*** **ED only** Post: *M*_*Diff*_ = 0.46/1.90 6-mo:*M*_*Diff*_ = 0.52/1.27 1-yr: *M*_*Diff*_ = 0.40/2.26 **ED + TA** ***vs*** **No ED** Post: ***M***_***Diff***_** = **− **1.41**/4.39 6-mo:***M***_***Diff***_** = **− **1.21**/**5.30** 1-yr: ***M***_***Diff***_** = **− **1.69**/**9.26** **ED only** ***vs*** **No ED** Post: ***M***_***Diff***_** = **− **0.95**/2.48 6-mo:*M*_*Diff*_ = − 0.68/4.02 1-yr: ***M***_***Diff***_** = **− **1.29**/**7.00**	**ED + TA ~ ED only** *ns:* (*p*-values unreported) **ED + TA > No ED** **PSFS: (** ***p*** **< .05)** Post, 6-months, 1-year **ODI: (** ***p*** **< .05)** 6-months, 1-year **ED only > No ED** **PSFS: (** ***p*** **< .05)** Post, 1-year **ODI: (** ***p*** **< .05)** 1-year *Effect sizes unreported*
[45] (2017) RCT (2 × 2) [Good]	Manipulating patient’s pain expectations using an inert solution/labelling, verbal instructions, and classical conditioning (CC)	Variation of CFs: Opioid Instruction (OI) (Deceptive/Hidden) **With CC**: (*n* = 12) **No CC**: (*n* = 12) Placebo Instruction (PI) (Truthful/Open-Label) **With CC**: (*n* = 12) **No CC**: (*n* = 12)	*Post: (Day 1)* Opioid Instruction **With CC:** *M* = 1.92 (± 1.73) **No CC**: *M* = 3.00 (± 2.73*)* Placebo Instruction: **With CC:** *M* = 4.58 (± 2.31) **No CC:** *M* = 5.83 (± 1.95)	**Deception > Truth** **(** ***p*** = < .01)* Dose–response: **Deception (OI)** With CC: *d* = 1.83* No CC: *d* = 0.83* **Truthful (PI)** With CC: *d* = 0.32; *ns* No CC: *d* = − 0.64* (**nocebo effect**)	*Post: (Day 1)* Opioid Instruction **With CC:** *M* = 77.22 (± 15.43) **No CC:** *M* = 67.78 (± 29.24) Placebo Instruction: **With CC:** *M* = 53.89 (± 24.03) **No CC:** *M* = 44.44 (± 15.66)	**Deception > Truth** **(** ***p*** = < .01)* Dose–response: **Deceptive: (OI)** With CC: *d* = − 0.92* No CC: *d* = − 0.59* **Truthful: (PI)** With CC: *d* = − 0.17 No CC: *d* = 0.43
[46] (2019) RCT (2 × 2) [Good]	Manipulating patient’s pain expectations using an inert drain dressing infusion with mirrors/labelling, verbal instructions, and either placebo or nocebo conditioning (PC or NC)	Variation of CFs: Sham “Opioid” Infusion: Placebo Conditioning (**PC**): (*n* = 17) Sham only (**SO**): (*n* = 21) Nocebo Conditioning (**NC**): (*n* = 21) Natural History (**NH**): (*n* = 14) no sham infusion (waiting only) nor any classical conditioning	*Post infusion: (Day 1)* **PC**: *M* = 3.24 (± 2.48) **SO**: *M* = 2.43 (± 1.88) **NC**: *M* = 3.57 (± 2.27) **NH**: *M* = 5.00 (± 2.35) *Post infusion: (Day 8)* **PC**: *M* = 3.41 (± 2.52) **SO**: *M* = 2.57 (± 2.22) **NC**: *M* = 3.48 (± 2.18) **NH**: *M* = 5.36 (± 1.98)	**Sham Infusion > NH** **PC & SO (** ***p*** **= < .001)** **NC (** ***p*** **= < .01)** **PC***: η*^*2*^ = 0.38 **SO***: η*^*2*^ = 0.56 **NC***: η*^*2*^ = 0.21 **NH: (** ***p*** **= .92)** **NH***: η*^*2*^ = 0.01	*Post infusion: (Day 1)* **PC**: *M* = 72.54 (± 29.2) **SO**: *M* = 77.46 (± 21.4) **NC**: *M* = 73.33 (± 23.2) **NH**: *M* = 54.76 (± 23.7) *Post infusion: (Day 8)* **PC**: *M* = 76.86 (± 29.2) **SO**: *M* = 78.73 (± 22.5) **NC**: *M* = 78.73 (± 18.0) **NH**: *M* = 53.86 (± 23.0)	**Sham Infusion > NH** **SO (** ***p*** **= < .01)** **PC & NC (** ***p*** **= < .05)** **PC***: η*^*2*^ = 0.15 **SO***: η*^*2*^ = 0.27 **NC***: η*^*2*^ = 0.20 **NH: (** ***p*** **= .63)** **NH***: η*^*2*^ = 0.03
[47] (2017) CCT [Excellent]	Enhanced Transtheoretical Model Intervention **(ETMI)** (*n* = 94): focusing on therapists’ communication skills; improving TA using empathy, active listening; addressing low motivation, self-efficacy, and addressing maladaptive beliefs/behaviours	Conventional physical therapy (**PT** (*n* = 95)) treatments: mobilisation, manipulation, back exercises, postural training, back school, electrical stimulation, shortwave diathermy, cooling, and stretching	Average Pain Post: *M*Δ = 0.6 95% C.I. [− 0.2 to 1.4] Follow-up: *M*Δ = 0.9 95% C.I. [− 0.03 to 1.8] Worst Pain Post: *M*Δ = 0.10 95% C.I. [− 0.8 to 1.2] Follow-up*: M*Δ = 1.2 95% C.I. [0.05 to 2.3]	**ETMI ~ PT** Post: (*p* = .10) Follow-up: (*p* = .06) Worst pain **ETMI > PT** Post: (*p* = .70) Follow-up: **(*p* = .04)** *Effect size unreported*	Post*: M*Δ = 1.3, 95% C.I. [− 0.3 to 3.0] Follow-up*: M*Δ = 2.7 95% C.I. [0.9 to 4.5]	**ETMI ~ PT** Post: (*p* = .10) **ETMI > PT** Follow-up: **(*p* = .004)** *d* = 0.54
[48] (2012) CCT [Good]	Intervention (*n* = 93) focused on patient’s illness and treatment beliefs and their information needs	**TAU** (*n* = 95): Treatment as usual; inpatient musculoskeletal rehabilitation which is typically multimodal and multidisciplinary	**Intervention** *M* = 42.91 (± 21.50) **TAU** *M* = 42.26 (± 20.77)	**Intervention ~ TAU** (*p* = .319)	**Intervention** *M* = 30.98 (± 15.70) **Control** *M* = 31.46 (± 16.19)	**Intervention ~ TAU** (*p* = .412)
[49] (2018) CCT [Fair]	Adding one weekly group-based physical therapy session in a rehabilitation setting compared to home treatment alone	Variation of CFs: **Rehab** (*n* = 13): Weekly group-based physical therapy session involving exercises **Home** (*n* = 17): No physical therapy supervision	*Post-treatment* *M*_*Diff*_ = − 0.9 95% C.I. [− 2.3 to 0.5]	**Rehab ~ Home** (*p* = .655)	*Post-treatment* *M*_*Diff*_ = − 0.2 95% C.I. [− 3.8 to 3.3]	**Rehab ~ Home** (p > 0.999)

* ~ indicates equivalence between groups; ns*: not statistically significant; 95% C.I.: 95% Confidence Interval; TUG: Timed-Up-and-Go (measured in seconds); RMDQ: Roland–Morris Disability Questionnaire – where higher scores represent higher levels of physical disability. PSFS: Patient-Specific Functional Scale – where higher scores represent higher levels of functioning; ODI: Oswestry Disability Index – where higher scores represent higher levels of physical disability

^a^This RCT involved three arms, including an open-label placebo (OLP) group. However, the OLP involved the administration of an injection rather than pills/capsules. These results were therefore excluded from the synthesis since it is an invasive procedure (exclusion criteria) and was not directly comparable to the other OLP trials

^b^Sex moderated the effect. Women in the CONNECT arm improved 4.94 RMDQ points compared to women in the control group

### Impact of contextual factors by type

Table 5 provides an overview of types of CF interventions and their impact on patient outcomes.

**Table 5:** Summary of Contextual Factor intervention types and their influence on patient outcomes

Ref no. (year) & Design	No. of CFs	Which CFs? (frequency)	How CFs manipulated during the intervention?	Influence on cLBP outcome(s)	Compared to active treatment	Effect size(s)
[36] (2013) RCT	1	Patient’s beliefs (1)	**Cognitive Behavioural Approach** – reframing back pain, explaining biopsychosocial pain mechanisms, changing maladaptive (i.e., fear-avoidant) movement using, goal setting, graded activity, and reflective communication	**Significant** improvement (*pain intensity* & *physical functioning*)	**Superior**	*Unreported*
[37] (2014) RCT (2 × 2)	2	Patient-practitioner relationship (1)	**Therapeutic Alliance** – interactions enhanced through verbal behaviours, active listening, tone of voice, non-verbal behaviours (i.e., eye contact, touch), and empathy	**Clinically meaningful** improvement (*pain intensity*)	**Superior** to limited TA	Sham + TA > Sham ***d* = 1.73** Active + TA > Active ***d* = 1.36**
		Treatment characteristics (1)	**Sham vs Active Treatment** – both patients and practitioners could not visually discriminate between sham or active IFC		**Superior** to Sham IFC	Active + TA > Sham + TA ***d* = 1.0** Active > Sham ***d* = 0.89**
[38] (2022) RCT	1	Patient’s beliefs (2)	**Cognitive-Behavioural and Affective Approach—**aims to shift patients’ beliefs about the causes and threat value of pain, focuses on reframing pain sensations through a lens of safety, addressing emotional threats and enhancing positive feelings and sensations through exposure to feared movements and evidence to provide reassurance	**Clinically meaningful** improvement (*pain intensity*) Improvement (*physical functioning*)	**Superior**	Pain Intensity ***g* = − 1.75** Physical Functioning ***g* = − 1.70**
[39] (2019) RCT	2	Patient’s beliefs (3)	**Implicit Cognitive Approach** – Verbal suggestions to positively influence patient’s symptom change expectations introduced by principal investigator wearing a white coat. **Social learning** – News report video (German subtitles/dubbing) regarding patients’ experiences of OLP to infer it is a legitimate/credible treatment approach	Improvement (*pain intensity* & *physical functioning*)	**Superior**	Pain Intensity ***d* = –0.44** Physical Functioning ***d* = –0.45**
		Treatment characteristics (2)	**Response Expectancy** – physical cues (i.e., typical, labelled medicine bottle and capsules) to connote pain-relieving treatment properties			
[40] (2016) RCT	2	Patient’s beliefs (4)	**Implicit Cognitive Approach** – Verbal suggestion to positively influence patient’s symptom change expectations using a warm and supportive communication style. **Social learning** – video of a news report regarding patients’ experiences of OLP to infer it is a legitimate/credible treatment approach	**Much Improved** (*pain intensity* & *physical functioning*)	**Superior**	Pain Intensity ***g* = 0.76** Physical Functioning ***g* = 0.74**
		Treatment characteristics (3)	**Response Expectancy** – physical cues (i.e., typical, labelled medicine bottle and capsules) to connote pain-relieving treatment properties			
[42] (2010) RCT	2	Patient’s beliefs (5)	**Explicit Cognitive Strategy –** Pain neuro-biology education (PNE) targeted misconceptions about the mechanisms of pain experiences (1 × 2.5 h)	**Significant** improvement (*pain intensity only*)	**PNE Superior** to PNE plus Exercise	*Unreported* **Note**: Patients attending group exercise classes interacted with non-trial staff/patients which may have undermined the PNE
		Patient-practitioner relationship (2)	**Additional Interactions –** group-based physical exercise classes open to the general community (via NHS)			
[45] (2017) RCT (2 × 2)	2	Patient’s beliefs (6)	**Implicit Cognitive Approach –** *Truthful* [or Deceptive] verbal suggestions to influence patient’s symptom change expectations: “*this solution is neutral, a placebo* [an opioid]*, it has no effect* [reduces pain and improves physical capacity]”)	**Significant** improvement (*pain intensity* & *physical functioning*)	**Superior** to truthful verbal suggestions	Pain Intensity With CC: ***d* = 1.83** No CC: ***d* = 0.83** Physical Functioning: With CC: ***d* = **− **0.92** No CC: ***d* = **− **0.59**
		Treatment characteristics (4)	**Response Expectancy** – visual and physical cues to connote pain-relieving treatment properties (i.e., bottles labelled as “*Opioid Klinische Prüfung”* (i.e., Opioid Clinical Trial). **Classical Conditioning** (CC) – 6 × experimental pain stimuli			
[46] (2019) RCT (2 × 2)	2	Patient’s beliefs (7)	**Implicit Cognitive Approach –** Deceptive verbal suggestions to influence patient’s symptom change expectations: “…*a new and very powerful transdermal infusion which reduces clinical back pain and improves functional capacity.*”)	**Significant** improvement (*pain intensity* & *physical functioning*)	**Superior** to Natural History group	Pain Intensity Sham Only*: ****η***^***2***^** = 0.56** Placebo Cond*: ****η***^***2***^** = 0.38** Nocebo Cond*: ****η***^***2***^** = 0.21** Physical Functioning: Sham Only*: ****η***^***2***^** = 0.27** Placebo Cond*: ****η***^***2***^** = 0.15** Nocebo Cond*: ****η***^***2***^** = 0.20**
		Treatment characteristics (5)	**Response Expectancy** – visual and physical cues to connote pain-relieving treatment properties (patch was labelled as “*Taroxin – hydromorphone, 1 mL* = *10 mg*, so patients believed it was a potent analgesic”), could see its application using mirrors and felt a damp sensation too. **Classical Conditioning** (CC) – use of experimental pain stimuli to positively (PC) or negatively (NC) influence pain perceptions			
[44] (2021) RCT	2	Patient’s beliefs (8)	**Explicit Cognitive Strategy –** Patient education (ED) relating to return to daily activities, advice on coping with pain, a clear explanation of signs and symptoms as recommended by treatment guidelines (2 × 1-h)	Improvement (*pain intensity* & *physical functioning*)	Equivalent *(ED* + *TA* ~ *ED only)* **Superior to No ED group**	Pain Intensity (1-year) *Unreported* Physical Functioning (see Table 4) *Unreported*
		Patient-practitioner relationship (3)	**Therapeutic Alliance** – In one group (ED + TA) the therapist aimed to enhance TA and empathy by emphasising a warm and caring reception, showing interest in the patient, asking about the patient’s condition in an interested manner, and demonstrating interest in the current complaint etc			
[35] (2011) RCT	3	Patient’s beliefs (9)	**Cognitive-Behavioural and Affective Approach** – including motivation enhancing factors such as proxy efficacy, treatment expectancy, and goal setting (MET)	**Significant** improvement (*pain intensity* & *physical functioning*)	Equivalent	*Not Applicable*
		Patient-practitioner relationship (4)	**Therapeutic Alliance** – use of motivational interviewing to develop working alliance			
		Treatment characteristics (6)	**Dummy MET** (Motivational Enhancement Treatment) – general communication skills, but deliberately avoided adopting MET-based counselling skills			
[43] (2020) RCT	2	Patient’s beliefs (10)	**Implicit Cognitive Approach** – Verbal suggestion to positively influence patient’s symptom change expectations (1-h session)	**Significant** improvement (*physical functioning* (RMDQ) *only*)	Equivalent	*Not Applicable*
		Treatment characteristics (7)	**Response Expectancy** – physical cues (i.e., typical, medicine bottle and capsules) to connote pain-relieving treatment properties			
[41] (2017) RCT (Cluster)	2	Patient’s beliefs (11)	**Explicit Cognitive Approach** – ensure patients understand their LBP and the relationship to physical activity; addressing fear-avoidance beliefs	Improvement (*pain intensity* & *physical functioning*)	Equivalent	**Note**: Sex moderated the effect. Women in the intervention arm improved (i.e., 4.94 RMDQ points lower) compared to women in the control arm
		Patient-practitioner relationship (5)	**Improved Communication** – enhance physiotherapists’ communication skills using the ‘5A’ framework (i.e., ask, advise, agree, assist, arrange)			
[47] (2017) CCT	2	Patient’s beliefs (12)	**Cognitive-Behavioural and Affective Approach** – address low motivation/self-efficacy for physical activity using behaviour change principles, graded activity to target fear-avoidance beliefs/behaviour, and educational messages informed by effective reassurance	Improvement (*pain intensity* & *physical functioning*)	Equivalent at post-treatment Superior at follow-up*	*Physical Functioning ***d* = 0.54**
		Patient-practitioner relationship (6)	**Therapeutic Alliance** – building the relationship with an emphasis on communicating empathy and active listening			
[48] (2012) CCT	1	Patient’s beliefs (13)	**Explicit Cognitive Strategy –** Educational intervention covering beliefs about medicines, rehabilitation, and individualised information to address unhelpful illness perceptions	**Significant** improvement (*pain intensity* & *physical functioning*)	Equivalent	**Note**: Control-arm involved in-patient multidisciplinary rehabilitation
[49] (2018) CCT	2	Patient-practitioner relationship (7)	**Additional Interactions –** one weekly group-based physical therapy session (i.e., extra time/attention)	**Significant** improvement (*pain intensity* & *physical functioning*)	Equivalent	*Not Applicable*
		Treatment Setting (1)	**Environment** – one group participated in physical therapy at home only whilst the other also attended weekly classes at a rehabilitation facility			
[50] (2015) Quasi-exp	1	Patient’s beliefs (14)	**Cognitive Behavioural Approach** – reframing back pain, explaining biopsychosocial pain mechanisms, changing maladaptive (i.e., fear-avoidant) movement using goal setting, graded activity, and reflective communication	**Significant** improvement (*pain intensity & physical functioning*)	*Not Applicable*	Pain Intensity ***d* = 0.65** Physical Functioning ***d* = 0.85**
[51] (2017) Quasi-exp	1	Patient’s beliefs (15)	**Explicit Cognitive Strategy –** Pain neuro-biology aimed at re-educating older patients on the relationship between LBP and normal aging processes	**Significant** improvement (*pain intensity* & *physical functioning*)	*Not Applicable*	Pain Intensity ***r* = 0.45** Physical Functioning partial ***η2* = 0.54**
[54] (2011) Obs. Cohort	1	Patient’s beliefs (16)	**Explicit Cognitive Strategy –** Using the Socratic dialogue technique to investigate and restructure patient’s maladaptive or unhelpful illness perceptions	**Significant** improvement (*physical functioning*)	*Not Applicable*	***r***^***2***^** = 3.9%** An increase in patient’s rational problem-solving skills was associated with improved physical functioning outcomes
**No direct manipulation of CFs**						
[52] (2013) Obs. Cohort	1	Patient-practitioner relationship (8)	***Measuring* Communication Skills** – patient information, perceived involvement in care, trust, satisfaction, and aspects of their communication behaviour during multimodal orthopaedic pain rehabilitation involving educational, psychotherapeutic, social, and occupation-related therapy	**Significant** improvement (*pain intensity* & *physical functioning*)	*Not Applicable*	Pain Intensity Post: ***d* = 0.60** Follow-up: ***d* = 0.48** Physical Functioning Post: ***d* = 0.53** Follow-up: ***d* = 0.48**
[53] (2013) Obs. Cohort	1	Patient-practitioner relationship (9)	***Measuring* Therapeutic Alliance –** sense of collaboration, warmth, and support between the patient and therapist. Includes agreement on (a) goals, (b) treatment, and (c) the affective or emotional bond	**Significant** improvement (*pain intensity* & *physical functioning*)	*Not Applicable*	One unit increase in TA reduced pain intensity by 0.044 units One unit increase in TA reduced disability by 0.113 units
[55] (2019) Obs. Cohort	1	Patient’s beliefs (17)	***Measuring relationships* between patients’ Competence Perceptions and Motivation** for undertaking physical therapy and whether patient motivations mediate the relationship between Competence Perceptions (CP) and pain and disability. *Competence Perceptions refers to the patient’s beliefs regarding their ability, efficacy, and proficiency to meet rehabilitation demands. Along a continuum, amotivation represents the least self-determined type whereas autonomous motivation is the most self-determined*	**Significant** associations (*pain intensity* & *physical functioning*)	*Not Applicable*	**Note**: Higher CP levels were associated with lower pain and disability at post-treatment Amotivation was the only significant mediator CP negatively predicted amotivation, which in turn positively predicted greater pain and disability

#### Patient’s beliefs and characteristics

Sixteen studies involved direct manipulation of patient’s beliefs and can be categorised according to their theoretical underpinnings which range from purely cognitive (i.e., both implicit and explicit), a combined cognitive-behavioural strategy, to those involving cognitive-behavioural and affective components. Eleven studies primarily aimed to address LBP-related fear-avoidance beliefs and associated behaviours, and/or maladaptive cognitions related to persistent LBP illness perceptions, pain mechanisms, and treatment [35, 36, 38, 41, 42, 44, 47, 48, 50, 51, 54] whilst five involved implicit learning/pre-cognitive associations [39, 40, 43, 45, 46] such as verbal suggestions. Overall, across the CF-intervention arms targeting patient’s beliefs, there is consistent evidence to suggest that altering cLBP illness or treatment perceptions positively influenced pain intensity (i.e., 7 RCTs [35, 36, 38, 40, 42, 45, 46], 1 CCT [48], 2 quasi-experimental studies [50, 51]; *n* = 837) and physical functioning (6 RCTs [35, 36, 40, 43, 45, 46], 1 CCT [48], 2 quasi-experimental studies [50, 51]; *n* = 751) outcomes. Six of the 16 studies modified patient’s beliefs alone [36, 38, 48, 50, 51, 54]; of these, both pain intensity and physical functioning substantially improved in five [36, 38, 48, 50, 51]. A cohort study (*n* = 135) which targeted unhelpful patient beliefs during treatment demonstrated an increase in patient’s rational problem-solving abilities predicted decreased disability (pain intensity was not an outcome) [54]. Another observational cohort (*n* = 64) measured the relationship between patient’s competence perceptions (beliefs regarding their ability to meet physical therapy demands) and found higher levels were associated with lower pain intensity and disability following rehabilitation [55]. Below is an overview of the different strategies used to modify patients’ beliefs and the corresponding results are summarised in Table 5.

##### Implicit cognitive strategies

are designed to tacitly or subtly influence patient’s expectations of an imminent symptom change either positively (e.g., anticipate less pain), negatively (e.g., anticipate more pain) or neutrally (e.g., anticipate no change). Five RCTs overtly targeted patients’ beliefs using verbal suggestions to influence patient’s expectations of symptom change (e.g., “*the placebo effect is powerful, and the body can automatically respond to placebo pills”* [40]). Three involved the administration of OLPs [39, 40, 43], and two combined this with a social learning approach [39, 40] using a video of a news report of other patient’s positive experiences of OLP to infer it is a legitimate treatment. One OLP trial reinforced the message midway through the trial [40] and reported both interactions were conducted in a warm and supportive manner. The other two RCTs used a sham opioid [45, 46] suggesting it would reduce pain and improve physical functioning (in the hidden/deception condition [45]).

##### Explicit cognitive strategies

aim to actively educate or alter patient’s LBP beliefs by targeting illness or treatment misconceptions/fallacies and/or provide accurate knowledge regarding pain modulation mechanisms. Two studies involved pain neuro-biology education interventions [42, 51]. Both targeted misconceptions about the mechanisms of pain experiences and used educational strategies to alter patient’s understanding of LBP. Whilst another two studies utilised Leventhal’s Common-Sense Model (CSM)/Self-regulation model as a theoretical basis to facilitate a change in patient’s illness and treatment perceptions [48, 54]. The CSM is a framework linking patients’ illness perceptions to behaviour and health outcomes. Lastly, although the primary focus of CONNECT trial [41] was augmenting the patient-practitioner relationship via enhanced communication skills, a sub-component involved addressing fear-avoidance beliefs via reshaping patient’s understanding of the relationship between pain and physical activity.

##### Cognitive-behavioural strategies

included interventions exclusively designed and tailored for persistent LBP combined with cognitive-behavioural principles (e.g., cognitive reframing, graded activity, goal setting). Two studies [36, 50] used Cognitive Functional Therapy (CFT) which is a bespoke intervention specifically designed for disabling LBP. CFT aims to normalise provocative movements while discouraging pain behaviours via cognitive reconceptualization, graded activity, and goal setting [58]. CFT appears to be the most arduous of the interventions for practitioners, considering 106 h of training was undertaken prior to its implementation [36].

##### Cognitive-behavioural and affective strategies

Contain elements of behaviour change techniques but also considers the patient’s emotional or affective state during rehabilitation. Two studies [35, 47] considered each patient’s initial state of motivation, as classified via the transtheoretical model (TTM; ‘stages-of-change’), and then used motivational interviewing (MI) to address patient’s beliefs, feelings, and behaviour [35, 47]. Whereas the PRT [38] trial aimed to shift patients’ beliefs about the causes and threat value of their pain experiences, by reframing pain sensations through a lens of safety, addressing emotional threats, along with gradual exposure to feared movements. PRT also incorporated pain neuro-biology education and aimed to consistently reinforce the same message throughout treatment [38].

#### Patient-Practitioner Relationship

Seven studies involved the direct modulation of the patient-practitioner relationship [35, 37, 41, 42, 44, 47, 49], whilst two observational cohorts [52, 53] measured aspects of the pre-existing dyadic relationship rather than purposefully altering it. These interventions are sub-categorised as follows: (2.1.) therapeutic alliance (TA:- creating a sense of collaboration, warmth, and support via technical skill, communicative competence, and reflective capacity) [35, 37, 44, 47, 53]; (2.2.) improved communication skills [41, 52]; and (2.3.) additional therapeutic interactions (i.e., extra attention/time) [42, 49]. There is some preliminary evidence (2 RCTs [35, 37], 1 CCT [47]*; n* = 413) that enhancing TA resulted in improved clinical outcomes from baseline, but there is an inconsistency since one study found no between-group differences after attempting to emphasise TA during two educational sessions [44]. The authors noted it was possible their attempts to improve TA failed, or perhaps a high level of TA was present after first contact with the patient regardless of group allocation [44]. Notably, these interventions all involved multiple components of care: physical (active treatments), cognitive (patient’s beliefs), and interpersonal (TA)—consequently, the impact of TA alone remains unclear. Only two of eight studies examined the role of the patient-practitioner relationship alone—both observational cohorts (*n* = 928). These indicated positive communication/relationship predicted improved pain intensity and physical functioning in patients with cLBP [52, 53]. Below is an overview of the different strategies used to influence the patient-practitioner relationship and the corresponding results are summarised in Table 5.

##### Therapeutic Alliance (TA)

Two interventions using Motivational Interviewing (MI) [35, 47] supported the development of TA by cultivating a sense of mutual collaboration between patients and practitioners using empathy and active listening. Although MI aims to facilitate a change in patient’s beliefs, the technique also involves fostering TA between the patient and practitioner by: (i) expressing accurate empathy, (ii) developing discrepancy, (iii) avoiding argumentation, and (iv) supporting patient’s self-efficacy. In a three-armed RCT [44], one group received an educational intervention with an emphasis on improving empathy and TA by providing a warm and caring reception, showing interest in the patient, and demonstrating interest in their complaint. In another RCT comparing enhanced versus limited TA [37], patients received enhanced TA through extra time to convey empathy, warmth, encouragement, and support. Irrespective of electrotherapy condition (active or sham), the enhanced TA patients had significantly larger improvements in pain intensity after a single session. Likewise, in an observational cohort, higher TA ratings at the end of the second treatment session were associated with significant decreases in both pain and disability outcomes [53].

##### Improved Communication Skills

The focus of the CONNECT trial was improving the patient-practitioner relationship via enhanced communication skills based on self-determination theory [41]. The intention was to facilitate the development of patient’s autonomy (i.e., feeling free to engage in activity), competence/self-efficacy (i.e., feeling effective or capable), and relatedness (i.e., feeling connected to and cared for by others) using the 5A framework. Eight hours of training positively influenced these physiotherapists’ communication skills, but independent observers rated their support below ideal (i.e., *M* = 4.57 using a 7-point rating scale) [41]. In an observational cohort study measuring various aspects the patient-practitioner relationship (i.e., trust, communication skills, and satisfaction with information received and expression of empathy), higher ratings on patient-practitioner variables were associated with improved pain and disability outcomes but inter-individual differences^1^ were apparent [52].

##### Additional Therapeutic Interactions (Attention/Time)

Two studies involved variations in time spent with the practitioner [42, 49]. In both studies the exercise classes were group-based, so it is unclear how much extra attention each patient received and whether there was continuity of care (i.e., same practitioner every class).

#### Treatment Characteristics

Seven RCTs involved a variation in the treatment characteristics either in terms of the absence or presence of the stimulus/cue/treatment condition [35, 37, 39, 40, 43, 45, 46]. Of these, five reported significant improvements in pain intensity following treatment (*n* = 409) [35, 37, 39, 40, 45, 46], whilst five of six reported significant improvements in physical functioning (*n* = 344) [35, 40, 43, 45, 46]. These studies involved administering sham/dummy treatments [35, 37], classical conditioning to manipulate pain perceptions [45, 46], or the presence/absence of visual or physical cues to denote pain-relieving treatment properties [39, 40, 43, 45, 46]. For example, during the application of a sham “opioid” infusion, the patch was labelled as “*Taroxin – hydromorphone, 1 mL* = *10 mg*” so patients believed it was a potent analgesic, they could see its application using mirrors, and also felt a damp sensation where applied [46]. Active treatments (namely IFC: interferential current therapy and MET: *Motivational Enhancement Treatment*), the presence of a medicalised symbolic cue (specifically an inert solution/infusion/capsules) or classical conditioning had a positive impact on both pain and physical functioning in patients with cLBP, suggesting there is consistent evidence relating to varying the treatment characteristics. However, none of these studies manipulated the treatment characteristics alone, since all these interventions involved more than one CF.

#### Therapeutic setting/environment

Only one study involved the manipulation of the therapeutic setting [49]. The principal difference between the two non-randomised groups were: one received weekly supervision from a physical therapist at the rehabilitation site, the other used an exercise booklet at home. All patients experienced improved clinical outcomes following the intervention but there were no significant between-group differences. This study had the lowest quality assessment grade (‘Fair’) across the studies but was adequately powered despite its small sample size (*n* = 30).

#### Practitioner’s beliefs and characteristics

None of the included studies modified practitioner beliefs or characteristics as a means of eliciting placebo analgesia in patients with cLBP.

## Discussion

### Summary of findings

Therapeutic encounters consist of multiple elements, the most obvious of which is an assumed specific treatment. These elements during clinical encounters, perceived as non-specific or implicit in nature—and referred to as CFs—may have important impacts on the modulation of pain and disability [11, 12]. The findings from this review suggest preliminary evidence for CFs adjunctive role and adds three unique contributions to the complex phenomenon of cLBP treatment.

Firstly, most patients with cLBP experienced improved clinical outcomes regardless of treatment arm. Overall, patients in the CF-manipulation arm(s) tended to demonstrate larger symptom improvements from baseline, even if the between-group differences were non-significant. There is initial evidence indicating CF-interventions appear, to some extent, comparable or equivalent to usual care/active treatments. CFs appear to be influencing both pain intensity and physical functioning outcomes over time in patients with cLBP. Since nearly all the included studies involved active treatments/comparison groups, and only two employed a no treatment/natural history group [44, 46], it is difficult to discern the precise level of impact of CFs on these outcomes compared to other confounders such as regression towards the mean. Pragmatic research designs were used as studies occurred in everyday rehabilitation settings, but findings may have differed if more of the studies included a waiting-list control. Of the two studies which included a no treatment condition, one was brief (8-days) [46], whilst the other only reported significant post-treatment between-group differences for one of the two disability measures (PSFS but not the ODI) [44]. In a series of neuroimaging studies, preliminary evidence suggested inactive pills successfully induced placebo analgesia that could not be explained by regression towards the mean, natural history, or mere exposure to the study [59]. To better disentangle effects underpinned by CFs, specific treatments, and natural history or regression to the mean, future studies might consider at least three comparison groups, including a waiting-list control (with the option of treatment at a later date), or factorial designs with a no treatment condition as this will enable a direct comparative view of the magnitude of any observed effects [60, 61].

Secondly, there is consistent evidence to indicate CF-manipulations may augment usual care treatment in rehabilitation settings in patients with cLBP. In studies with at least two comparison groups [35–49], half reported significant improvements in pain intensity, in favour of the CF-interventions [36–40, 42, 45, 46]. Notable CFs influencing pain intensity outcomes included (a) patient-centred education to address misinformed, unhelpful, or maladaptive cLBP or pain-related beliefs (i.e., *illness representations*); (b) verbal suggestions to influence patient's symptom change beliefs (i.e., *treatment expectations*); (c) visual or physical cues (i.e., *treatment characteristics*) to connote pain-relieving treatment properties (i.e., *treatment expectations*); and (d) positive or patient-centred communication to promote the therapeutic alliance (i.e., *patient-practitioner relationship*).

Similarly, half the studies demonstrated significant improvements in favour of the CF-manipulation arm(s) for physical functioning outcomes [36, 38–40, 44–46]. The same CFs were apparent, with a few variations regarding the patient-practitioner relationship. For instance, facilitating TA via reassurance was only significant at 12-months’ follow-up, not post-treatment [47], and female patients were more responsive to an intervention enhancing communication and TA than males [41]. This review found the strongest evidence relates to patient’s expectations/beliefs. When reported, the magnitude of effects was generally medium to large, suggesting these CFs had a meaningful impact on clinical outcomes despite their heterogeneity. The findings were less consistent for the patient-practitioner relationship; although enhancing TA appears to be influential, the best approach for achieving an improved working relationship may require further training, such as motivational interviewing.

Treatment expectation shapes the patient's pain experience [62–64] which is a recognised prognostic factor in MSK pain [65–67]. A patient's prior treatment experiences and preferences can also affect the outcome [68] and alter the magnitude of the response in MSK rehabilitation [69]. General expectations for pain relief influence pain and physical functioning in patients with LBP [70, 71] and neck pain [72] as well as practitioner-rated outcome expectancies [73, 74]. Ignoring patients’ preferences, expectations, or prior experiences can negatively influence outcomes [75]. A meta-analysis involving interventions which aimed to induce expectation, using verbal suggestion, conditioning, or mental imagery on patient’s pain indicated the effects on chronic pain were small [64]. It suggested that combining different forms of expectations and more extensive interventions that addressed the patient’s expectations might enhance these effects which is consistent with the findings from this review.

The patient-practitioner relationship also positively influences outcomes like pain, physical functioning [73, 74], patient satisfaction, and strengthens the therapeutic alliance [76]. Empathy and expectation are notable features for reducing pain [77]. Both therapeutic alliance and practitioner-rated expectations of how each patient will respond to treatment were the strongest predictors of back-related disability in a prospective cohort study in a rehabilitation setting [74]. These effects were however mediated by improved patient self-efficacy in pain coping, perceiving back pain as less threatening, along with a reduction in psychosocial distress [74]. Similarly, a systematic review examining patient-practitioner communication found that increasing practitioner empathy and encouraging positive patient expectations had small but significant effects on acute pain [78]. Although heterogeneity between interventions made it difficult to pinpoint the effective elements. A variety of communication skills such as active listening, paraphrasing, language reciprocity, verbal encouragement, humour, and empathy have been shown to influence treatment outcomes [69, 75, 79, 80]. In this review, intensive training (e.g., CFT) seems to have had a stronger influence on patient outcomes compared to shorter training. The reason being that specialised psychosocial competences are not typically incorporated into undergraduate training programmes. It is suggested that the influence of the early acquisition of these skills is investigated in future.

Lastly, it is possible that modifying more than one CF may be more impactful on patients’ clinical outcomes. This review found consistent evidence relating to the treatment characteristics; but all seven RCTs involved more than one CF. It is therefore challenging to ascertain which CFs may have influenced overall clinical improvements and may be complicated by any synergistic action between CFs. The quality assessment highlighted that these innovative approaches may not have direct clinical utility, and there is considerable debate concerning the ethical application of ‘placebos’ which is intrinsically linked to definitional ambiguities [81] and their perceived illegitimacy historically [82]. For instance, the three OLP trials included in this review reported differing outcomes. The administration approaches were similar, but not identical, suggesting future studies might investigate patients’ experiences to understand how these cues are perceived and which are essential elements for reliably inducing placebo analgesia using OLP. In a study using an inert cream, placebo analgesia clearly increased in a “dose”-dependent manner, mediated by the anticipated level of pain-relief (i.e., corresponding to the degree of conditioned expectation) [83]. The authors [83] explained placebo analgesia as:

a dynamic product of interactions among expectations, physiological arousal, and somatic perception. Over time, the individual in pain inevitably evaluates how well his expectation of relief comports with reality, and this comparison can influence future expectation. Past success in decreasing pain increases the expectation that future relief is possible, while past failure suppresses the expectation of future success.

This illustrates the complex interplay between all five CFs; none are static states, rather dynamic, fluid synergies. Patients are continually interpreting and being influenced by co-occurring internal and external contexts and cues, including interpersonal interactions during health encounters, through the lens of their prior experiences, to anticipate if symptom change can be expected [11, 12]. It seems explicitly inducing placebo analgesia is informed by the cogency and consistency between the CFs (i.e., creating a credible and coherent ‘story’) to evoke this innate biological response. Modifying more than one CF may be more impactful on patients’ outcomes, namely: attempting to create coherence between illness representations and treatment expectations whilst ensuring consistency between treatment characteristics and treatment expectations; along with cultivating the patient-practitioner relationship.

In this sense, practitioners could be viewed as the “sugar pill”. What appears to be an important therapeutic process is the manner in which a practitioner interacts with their patient, such as expressing empathy and warmth, to facilitate the development of TA or a working relationship which might then enable practitioners to address misinformed or unhelpful cLBP illness beliefs negatively influencing patient’s cognitions and behaviour (e.g., vicious cycle of pain, fear-avoidance, catastrophising). Furthermore, practitioners might simultaneously aim to influence patient’s treatment expectations regarding symptom improvements through feedback (e.g., visual, or physical cues and/or verbal suggestions) to explain how or why the features of the conservative treatment are suitable or effective for the patient’s cLBP (i.e., to develop treatment credibility). These two processes may be clinically useful approaches which help explain the role of important CFs positively influencing pain intensity and physical functioning outcomes in those with cLBP.

### Strengths and limitations

This review used a robust search strategy evaluated by two experienced librarians. The array of search terms arising from the plethora of interchangeable terminology illustrates the need for an integrated theoretical framework [24]. Although Howick’s paper [26] helps to refine and clarify definitional issues, the chosen CF framework offered a utilitarian approach. It is plausible the inclusion–exclusion criteria precluded studies where practitioner’s beliefs/expectations or characteristics were overtly manipulated. An ineligible RCT, identified via the search strategy, involving 128 patients with acute, non-specific LBP patients found that formal or casual attire had no effect on treatment credibility [84]. Accordingly, the search strategy was sensitive and specific enough to identify studies which may have modified this CF, but none were eligible for inclusion. However, future research should examine the crucial role of practitioner’s beliefs/expectations and characteristics (see [85]). Most of the included studies were not specifically designed as CF-interventions but focusing on everyday treatment settings may enable the findings to be adapted for clinical use. The included studies utilised complex interventions, with multiple components, and modified one or more CFs making it difficult to separate out the precise influence of a specific CF (see [86] for a discussion).

This review may not be all-encompassing; grey literature, retrospective cohorts, and secondary analyses were excluded. There is potential bias as a single reviewer conducted the screening, data extraction, and quality assessment but a sample of the of potentially eligible full-text articles were independently cross-checked by the entire review team. Since the included studies were fairly heterogeneous, it may have been worthwhile using several quality assessment tools rather than modifying the scoring criteria. Overall, only one study was graded as ‘Fair’ but since eligible studies were published between 2009 and 2022, current reporting standards in conjunction with research checklists/guidelines may have influenced the quality of studies included. Key issues affecting quality related to statistical power and generalizability. Cumulative low scoring on item 11 (external validity) implies that these findings are not necessarily generalisable since both men and older patients are likely under-represented. These studies were also generally clustered in the Northern hemisphere and may overrepresent patients from developed or higher income countries. Similarly, studies scoring ‘0’ regarding the representativeness of the staff, facilities, intervention, and setting (item 13) used novel, bespoke, or innovative approaches to care. Although this is not necessarily problematical, it suggests that specific interventions may not have immediate practical utility, nor be directly transferable to other rehabilitation settings without appropriate modification. Consequently, these findings are promising, but require judicious interpretation.

## Conclusion

In conclusion, this systematic review has demonstrated preliminary evidence to indicate explicitly leveraging CFs augments conservative cLBP treatment. It identified CFs reducing pain intensity and improving physical functioning outcomes and extracted specific strategies with prospective clinical utility. The heterogeneity of interventions suggests modifying more than one CF may be more impactful. In essence, the practitioner’s therapeutic potency lies in their capacity to simultaneously provide physical, cognitive, and emotional care to influence the patient’s mindset and consequently their physiology.

## Supplementary Information

**Additional file 1**. The search strategies per database (**Methods S1-S4**), a scoring grid for item 11 of the quality assessment (**Results S1**), a summary of the study characteristics (**Table S1**), and a summary of the within group changes from baseline (**Table S2**).

## Data Availability

The dataset generated during and/or analysed during the current study are not publicly available yet since it will be published in Bournemouth University’s online research data repository (BORDaR) following the completion of the dissertation. It is available from the corresponding author on reasonable request and with the permission of Bournemouth University via a data sharing agreement.

## Abbreviations

MSK: Musculoskeletal
LBP: Low back pain
cLBP: Chronic low back pain
NSAID: Non-steroidal anti-inflammatory drug
CFs: Contextual factors
NICE: National Institute for Health and Care Excellence
TIDieR: Template of Intervention Description and Replication
EPOC: Cochrane Effective Practice and Organisation of Care Review Group
ESRC: Economic and Social Research Council
RCT: Randomised Controlled Trial
CCT: Controlled Clinical Trial
IFC: Interferential current therapy
NRS: Numeric Rating Scale
VAS: Visual Analogue Scale
ODI: Oswestry Disability Index
PSFS: Patient Specific Functional Scale
ADL: Hannover Activities of Daily Living Questionnaire
OLP: Open-label placebo
MCID: Minimal clinically important difference
CSM: Leventhal’s Common-Sense Model
CONNECT: Communication Style and Exercise Compliance in Physiotherapy
CFT: Cognitive Functional Therapy
TTM: Transtheoretical model (‘stages of change’)
MI: Motivational interviewing
MET: Motivational Enhancement Treatment
PT: Physical therapy
MT-EX: Manual therapy and exercise
TAU: Treatment as usual
ED-EX: Education and exercise
ETMI: Enhanced Transtheoretical Model Intervention
TA: Therapeutic alliance
PNE: Pain neuro-biology education
CC: Classical conditioning
PRT: Pain Reprocessing Therapy
ED: Patient education
ED + TA: Patient education and therapeutic alliance

## Footnotes

1 For instance, some patients had lower improvements if the practitioner was perceived to have communicated in a patient-centred manner and involved them in treatment decisions. These patients also tended to rate their satisfaction and trust in their practitioner lower too, suggesting patient-centred communication ought to consider patient’s particular preferences (e.g., collaborative versus authoritative) or possibly their characteristics.
