# Supplementary material for: Impact of contextual factors on patient outcomes following conservative low back pain treatment: systematic review
Source: Chiropr Man Therap. 2022 Apr 21;30:20. doi: 10.1186/s12998-022-00430-8 (PMC9028033; doi:10.1186/s12998-022-00430-8)
Supplement: Supplementary file 1 — Additional file 1. The search strategies per database (Methods S1-S4), a scoring grid for item 11 of the quality assessment (Results S1), a summary of the study characteristics (Table S1), and a summary of the within group changes from baseline (Table S2). [file 12998_2022_430_MOESM1_ESM.docx]

### List of Supplementary Information

**Methods S1.** Search Strategy for Medline

**Methods S2.** Search Strategy for CINAHL

**Methods S3.** Search Strategy for PsycINFO

**Methods S4.** Search Strategy for AMED

**Results S1.** External validity sub-scale (Item 11): Quality assessment scoring grid

**Table S1.** Summary of study characteristics clustered by research design

**Table S2.** Contextual Factor intervention group(s) within-group change in outcomes from baseline clustered by research design

**Methods S1.** Search Strategy for Medline

| **Line Number** | **Search Terms** |
| --- | --- |
| 1 | (placebo ADJ (effect* OR response* OR analgesi*)).ti,ab |
| 2 | (nocebo ADJ (effect* OR response* OR hyperalgesia)).ti,ab |
| 3 | (context* ADJ (factor* OR effect* OR response*)).ti,ab |
| 4 | (common ADJ (factor* OR effect*)).ti,ab |
| 5 | (non?specific ADJ (effect* OR factor*)).ti,ab |
| 6 | (alliance*).ti,ab |
| 7 | (patient ADJ (relation* OR interact*)).ti,ab |
| 8 | (patient* ADJ (expect* OR belief* OR attitude*)).ti,ab |
| 9 | (practitioner* ADJ (belief* OR attitude* OR effect*)).ti,ab |
| 10 | (positive ADJ (expect* OR suggest*)).ti,ab |
| 11 | (negative ADJ (expect* OR suggest*)).ti,ab |
| 12 | (empath* OR warm* OR kind* OR compassion* OR friendl*).ti,ab |
| 13 | (rapport).ti,ab |
| 14 | (“open label placebo”).ti,ab |
| 15 | (expect* ADJ (effect* OR response*)).ti,ab |
| 16 | (patient* ADJ (experience* OR perspective*)).ti,ab |
| 17 | (illness ADJ (perception* OR belief*)).ti,ab |
| 18 | (“initial consultation”).ti,ab |
| 19 | (“white coat” OR “white?coat effect”).ti,ab |
| 20 | exp “NOCEBO EFFECT”/ |
| 21 | exp “PLACEBO EFFECT”/ |
| 22 | exp “THERAPEUTIC ALLIANCE”/ |
| 23 | exp “PROFESSIONAL-PATIENT RELATIONS”/ |
| 24 | exp “PHYSICIAN-PATIENT RELATIONS”/ |
| 25 | exp HOPE/ |
| 26 | exp “FACIAL EXPRESSION”/ |
| 27 | exp “NONVERBAL COMMUNICATION”/ |
| 28 | exp “VERBAL BEHAVIOR”/ |
| 29 | exp “PERSUASIVE COMMUNICATION”/ |
| 30 | exp “HEALTH COMMUNICATION”/ |
| 31 | exp “FACIAL EXPRESSION”/ |
| 32 | exp “ATTITUDE OF HEALTH PERSONNEL”/ |
| 33 | exp TRUST/ |
| 34 | (“back pain”).ti,ab |
| 35 | (“low back pain”).ti,ab |
| 36 | (“LBP”).ti,ab |
| 37 | (“chronic low back pain”).ti,ab |
| 38 | (“cLBP”).ti,ab |
| 39 | (“persistent low back pain”).ti,ab |
| 40 | (“non?specific low back pain”).ti,ab |
| 41 | (“non?specific back pain”).ti,ab |
| 42 | (“lumbar pain”).ti,ab |
| 43 | (lumbago).ti,ab |
| 44 | (radiculitis).ti,ab |
| 45 | (sciatica).ti,ab |
| 46 | (“discogenic low back pain”).ti,ab |
| 47 | (“facet joint pain”).ti,ab |
| 48 | (“sacroiliac joint pain”).ti,ab |
| 49 | exp SCIATICA/ |
| 50 | exp “LUMBOSACRAL REGION”/ |
| 51 | exp “LOW BACK PAIN”/ |
| 52 | exp “BACK PAIN”/ |
| 53 | (1 OR 2 OR 3 OR 4 OR 5 OR 6 OR 7 OR 8 OR 9 OR 10 OR 11 OR 12 OR 13 OR 14 OR 15 OR 16 OR 17 OR 18 OR 19 OR 20 OR 21 OR 22 OR 23 OR 24 OR 25 OR 26 OR 27 OR 28 OR 29 OR 30 OR 31 OR 32 OR 33) |
| 54 | (34 OR 35 OR 36 OR 37 OR 38 OR 39 OR 40 OR 41 OR 42 OR 43 OR 44 OR 45 OR 46 OR 47 OR 48 OR 49 OR 50 OR 51 OR 52) |
| **59** | (53 AND 54) *[DT 2009-2022]* |

**Methods S2.** Search Strategy for CINAHL

| **Line Number** | **Search Terms** |
| --- | --- |
| 1 | (placebo ADJ (effect* OR response* OR analgesi*)).ti,ab |
| 2 | (nocebo ADJ (effect* OR response* OR hyperalgesia)).ti,ab |
| 3 | (context* ADJ (factor* OR effect* OR response*)).ti,ab |
| 4 | (common ADJ (factor* OR effect*)).ti,ab |
| 5 | (non?specific ADJ (effect* OR factor*)).ti,ab |
| 6 | (alliance*).ti,ab |
| 7 | (patient ADJ (relation* OR interact*)).ti,ab |
| 8 | (patient* ADJ (expect* OR belief* OR attitude*)).ti,ab |
| 9 | (practitioner* ADJ (belief* OR attitude* OR effect*)).ti,ab |
| 10 | (positive ADJ (expect* OR suggest*)).ti,ab |
| 11 | (negative ADJ (expect* OR suggest*)).ti,ab |
| 12 | (empath* OR warm* OR kind* OR compassion* OR friendl*).ti,ab |
| 13 | (rapport).ti,ab |
| 14 | (“open label placebo”).ti,ab |
| 15 | (expect* ADJ (effect* OR response*)).ti,ab |
| 16 | (patient* ADJ (experience* OR perspective*)).ti,ab |
| 17 | (illness ADJ (perception* OR belief*)).ti,ab |
| 18 | (“initial consultation”).ti,ab |
| 19 | (“white coat” OR “white?coat effect”).ti,ab |
| 20 | exp "PLACEBO EFFECT"/ |
| 21 | exp PLACEBOS/ |
| 22 | exp "PROFESSIONAL-CLIENT RELATIONS"/ |
| 23 | exp "PHYSICIAN-PATIENT RELATIONS"/ |
| 24 | exp "NURSE-PATIENT RELATIONS"/ |
| 25 | exp "PROFESSIONAL-PATIENT RELATIONS"/ |
| 26 | exp "INTERPERSONAL RELATIONS"/ |
| 27 | exp "VERBAL BEHAVIOR"/ |
| 28 | exp "NONVERBAL COMMUNICATION"/ |
| 29 | exp "COMMUNICATION SKILLS"/ |
| 30 | exp COMMUNICATION/ |
| 31 | exp TRUST/ |
| 32 | exp CHARACTER/ |
| 33 | exp CARING/ |
| 34 | exp EMPATHY/ |
| 35 | exp "THERAPEUTIC ALLIANCE"/ |
| 36 | exp COMPASSION/ |
| 37 | exp HOPE/ |
| 38 | exp "ATTITUDE OF HEALTH PERSONNEL"/ |
| 39 | (“back pain”).ti,ab |
| 40 | (“low back pain”).ti,ab |
| 41 | (“LBP”).ti,ab |
| 42 | (“chronic low back pain”).ti,ab |
| 43 | (“cLBP”).ti,ab |
| 44 | (“persistent low back pain”).ti,ab |
| 45 | (“non?specific low back pain”).ti,ab |
| 46 | (“lumbar pain”).ti,ab |
| 47 | (sciatica).ti,ab |
| 48 | (“discogenic low back pain”).ti,ab |
| 49 | exp "LOW BACK PAIN"/ |
| 50 | exp "BACK PAIN"/ |
| 51 | (1 OR 2 OR 3 OR 4 OR 5 OR 6 OR 7 OR 8 OR 9 OR 10 OR 11 OR 12 OR 13 OR 14 OR 15 OR 16 OR 17 OR 18 OR 19) |
| 52 | (20 OR 21 OR 22 OR 23 OR 24 OR 25 OR 26 OR 27 OR 28 OR 29 OR 30 OR 31 OR 32 OR 33 OR 34 OR 35 OR 36 OR 37 OR 38) |
| 53 | (51 OR 52) |
| 54 | (39 OR 40 OR 41 OR 42 OR 43 OR 44 OR 45 OR 46 OR 47 OR 48 OR 49 OR 50) |
| 55 | (53 AND 54) *[DT 2009-2022]* |

**Methods S3.** Search Strategy for PsycINFO

| **Line Number** | **Search Terms** |
| --- | --- |
| 1 | (placebo ADJ (effect* OR response* OR analgesi*)).ti,ab |
| 2 | (nocebo ADJ (effect* OR response* OR hyperalgesia)).ti,ab |
| 3 | (context* ADJ (factor* OR effect* OR response*)).ti,ab |
| 4 | (common ADJ (factor* OR effect*)).ti,ab |
| 5 | (non?specific ADJ (effect* OR factor*)).ti,ab |
| 6 | (alliance*).ti,ab |
| 7 | (patient ADJ (relation* OR interact*)).ti,ab |
| 8 | (patient* ADJ (expect* OR belief* OR attitude*)).ti,ab |
| 9 | (practitioner* ADJ (belief* OR attitude* OR effect*)).ti,ab |
| 10 | (positive ADJ (expect* OR suggest*)).ti,ab |
| 11 | (negative ADJ (expect* OR suggest*)).ti,ab |
| 12 | (empath* OR warm* OR kind* OR compassion* OR friendl*).ti,ab |
| 13 | (rapport).ti,ab |
| 14 | (“open label placebo”).ti,ab |
| 15 | (expect* ADJ (effect* OR response*)).ti,ab |
| 16 | (patient* ADJ (experience* OR perspective*)).ti,ab |
| 17 | (illness ADJ (perception* OR belief*)).ti,ab |
| 18 | (“initial consultation”).ti,ab |
| 19 | (“white coat” OR “white?coat effect”).ti,ab |
| 20 | exp PLACEBO/ |
| 21 | exp "THERAPEUTIC ALLIANCE"/ |
| 22 | exp "COMMON FACTORS"/ |
| 23 | exp "INTERPERSONAL RELATIONSHIPS"/ |
| 24 | exp "THERAPEUTIC ENVIRONMENT"/ |
| 25 | exp "CARING BEHAVIORS"/ |
| 26 | exp "INTERPERSONAL INTERACTION"/ |
| 27 | exp EXPECTATIONS/ |
| 28 | exp HOPE/ |
| 29 | exp "TRUST (SOCIAL BEHAVIOR)"/ |
| 30 | exp "LISTENING (INTERPERSONAL)"/ |
| 31 | exp "EYE CONTACT"/ |
| 32 | exp "BODY LANGUAGE"/ |
| 33 | exp "ACTIVE LISTENING"/ |
| 34 | exp "INTERPERSONAL COMMUNICATION"/ |
| 35 | exp "CLIENT ATTITUDES"/ |
| 36 | exp GESTURES/ |
| 37 | exp "FACIAL EXPRESSIONS"/ |
| 38 | exp "VERBAL LEARNING"/ |
| 39 | (“back pain”).ti,ab |
| 40 | (“low back pain”).ti,ab |
| 41 | (“LBP”).ti,ab |
| 42 | (“chronic low back pain”).ti,ab |
| 43 | (“cLBP”).ti,ab |
| 44 | (sciatica).ti,ab |
| 45 | exp "BACK PAIN"/ |
| 46 | (1 OR 2 OR 3 OR 4 OR 5 OR 6 OR 7 OR 8 OR 9 OR 10 OR 11 OR 12 OR 13 OR 14 OR 15 OR 16 OR 17 OR 18 OR 19) |
| 47 | (20 OR 21 OR 22 OR 23 OR 24 OR 25 OR 26 OR 27 OR 28 OR 29 OR 30 OR 31 OR 32 OR 33 OR 34 OR 35 OR 36 OR 37 OR 38) |
| 48 | (46 OR 47) |
| 49 | (39 OR 40 OR 41 OR 42 OR 43 OR 44 OR 45) |
| 50 | (48 AND 49) *[DT 2009-2022]* |

**Methods S4.** Search Strategy for AMED

| **Line Number** | **Search Terms** |
| --- | --- |
| 1 | (placebo ADJ (effect* OR response* OR analgesi*)).ti,ab |
| 2 | (nocebo ADJ (effect* OR response* OR hyperalgesia)).ti,ab |
| 3 | (context* ADJ (factor* OR effect* OR response*)).ti,ab |
| 4 | (common ADJ (factor* OR effect*)).ti,ab |
| 5 | (non?specific ADJ (effect* OR factor*)).ti,ab |
| 6 | (alliance*).ti,ab |
| 7 | (patient ADJ (relation* OR interact*)).ti,ab |
| 8 | (patient* ADJ (expect* OR belief* OR attitude*)).ti,ab |
| 9 | (practitioner* ADJ (belief* OR attitude* OR effect*)).ti,ab |
| 10 | (positive ADJ (expect* OR suggest*)).ti,ab |
| 11 | (negative ADJ (expect* OR suggest*)).ti,ab |
| 12 | (empath* OR warm* OR kind* OR compassion* OR friendl*).ti,ab |
| 13 | (rapport).ti,ab |
| 14 | (“open label placebo”).ti,ab |
| 15 | (expect* ADJ (effect* OR response*)).ti,ab |
| 16 | (patient* ADJ (experience* OR perspective*)).ti,ab |
| 17 | (illness ADJ (perception* OR belief*)).ti,ab |
| 18 | (“initial consultation”).ti,ab |
| 19 | (“white coat” OR “white?coat effect”).ti,ab |
| 20 | exp PLACEBOS/ |
| 21 | exp "NURSE PATIENT RELATIONS"/ |
| 22 | exp "PHYSICIAN PATIENT RELATIONS"/ |
| 23 | exp "PROFESSIONAL PATIENT RELATIONS"/ |
| 24 | exp "VERBAL BEHAVIOR"/ |
| 25 | exp "TRUTH DISCLOSURE"/ |
| 26 | exp "NONVERBAL COMMUNICATION"/ |
| 27 | exp COMMUNICATION/ |
| 28 | exp "INTERPERSONAL RELATIONS"/ |
| 29 | exp EMPATHY/ |
| 30 | exp "ATTITUDE OF HEALTH PERSONNEL"/ |
| 31 | (“back pain”).ti,ab |
| 32 | (“low back pain”).ti,ab |
| 33 | (“LBP”).ti,ab |
| 34 | (“chronic low back pain”).ti,ab |
| 35 | (“cLBP”).ti,ab |
| 36 | (“non?specific low back pain”).ti,ab |
| 37 | (sciatica).ti,ab |
| 38 | (“sacroiliac joint pain”).ti,ab |
| 39 | exp "LOW BACK PAIN"/ |
| 40 | exp BACKACHE/ |
| 41 | (1 OR 2 OR 3 OR 4 OR 5 OR 6 OR 7 OR 8 OR 9 OR 10 OR 11 OR 12 OR 13 OR 14 OR 15 OR 16 OR 17 OR 18 OR 19) |
| 42 | (20 OR 21 OR 22 OR 23 OR 24 OR 25 OR 26 OR 27 OR 28 OR 29 OR 30) |
| 43 | (41 OR 42) |
| 44 | (31 OR 32 OR 33 OR 34 OR 35 OR 36 OR 37 OR 38 OR 39 OR 40) |
| 45 | (43 AND 44) *[DT 2009-2022]* |

**Results S1.** External validity sub-scale (Item 11): Quality assessment scoring grid

| **Reference** | **Gender**  Ratio of females < 60.0% | **Age**  Mean Sample Age:  ≥ 40.00 and ≤ 63.5 years | **Q11 Decision**  (Score) |
| --- | --- | --- | --- |
| **RCT** | | | |
| [35] | No | Yes | No (0) |
| [36] | Yes | Yes | Yes (1) |
| [37] | No | No | No (0) |
| [38] | Yes | Yes | Yes (1) |
| [39] | No | Yes | No (0) |
| [40] | No | Yes | No (0) |
| [41] | Yes | Yes | Yes (1) |
| [42] | No | Yes | No (0) |
| [43] | No | No | No (0) |
| [44] | Yes | Yes | Yes (1) |
| [45] | No | Yes | No (0) |
| [46] | Yes | Yes | Yes (1) |
| **CCT** | | | |
| [47] | Yes | Yes | Yes (1) |
| [48] | No (unclear 5% missing) | Yes | No (0) |
| [49] | No | Not Specified | No (0) |
| **Quasi-Experimental** | | | |
| [50] | Yes | Yes | Yes (1) |
| [51] | Yes | No | No (0) |
| **Observational cohort** | | | |
| [52] | Yes | Yes | Yes (1) |
| [53] | No | Yes | No (0) |
| [54] | Yes | Yes | Yes (1) |
| [55] | No | Yes | No (0) |
| **Total** | **10 (47.62%)** | **17 (80.95%)** | **9 (42.86%)** |

**Table S1.** Summary of study characteristics clustered by research design

| **Reference** | **Country of Origin** | **Study Setting** | **Total Sample size (*n*)** | **Mean age**  **Years** (*SD*) | **Gender Proportions**  (F : M %) | **Contextual Factor(s)** | **Frequency / Duration of Contextual Factor Treatment** |
| --- | --- | --- | --- | --- | --- | --- | --- |
| **Randomised Controlled Trials** (RCTs) | | | | | | | |
| [35] | Not specified (possibly Hong Kong) | Local outpatient physical therapy department | Allocated (88)  Baseline (76)  Midpoint (63)  Post-Tx (60)  Follow-up (55) | Control:  45.1 (±10.7);  Intervention: 44.6 (±11.2) | 63% : 37% | 1) Patient’s beliefs;  3) Patient-practitioner relationship;  5) Treatment characteristics | - 10 individual / one-on-one sessions (± 30 minutes each) - Duration: 8 weeks |
| [36] | Norway | Three different private clinics | Allocated (121)  Baseline (121)  Post-Tx (94)  Follow-up (88) | Control:  42.9 (±12.5);  Intervention:  41.0 (±10.3) | 52% : 48% | 1) Patient’s beliefs | - Weekly: first 2 to 3 sessions; - Then one session every 2–3 weeks (12 weeks duration); - One-on-one sessions |
| [37] | Canada | University affiliated sports physical therapy/ rehabilitation centre | Allocated (117)  Baseline (117)  Post-Tx (117) | Combined:  30.0 (±6.8) | 60% : 40% | 3) Patient-practitioner relationship;  5) Treatment characteristics | - One individual / one-on-one session (± 30 to 45 minutes) |
| [38] | United States of America (USA, Colorado) | A University setting | Allocated (100)  Baseline (100)  Post-Tx (91)  1-month (79)  2-month (75)  3-month (74)  6-months (79)  12-months (81) | Control:  41.3 (±15.9);  Intervention: 42.6 (±16.2) | 56% : 44% | 1) Patient’s beliefs | - 8 individual / one-on-one sessions (± 60 minutes each) - Twice weekly for 4 weeks |
| [39] | Germany | Back Pain Centre, University Hospital | Allocated (127)  Baseline (122)  Post-Tx (122) | Control:  58.4 (±14.0);  Intervention: 60.3 (±15.2) | 62% : 38% | 1) Patient’s beliefs;  5) Treatment characteristics | - 21 days (capsules taken twice a day); - Video providing standardised information regarding the placebo effect before randomisation. |
| [40] | Portugal | Outpatient pain unit in a general public hospital | Allocated (97)  Baseline (83)  Post-Tx (76) | Control:  44.1 (±13.7);  Intervention:  44.4 (±13.2) | 71% : 29% | 1) Patient’s beliefs;  5) Treatment characteristics | - 21 days (2 pills twice a day) plus treatment as usual; - 2 x individual / one-on-one interactions with Principal investigator (10-15 minutes each) |
| [41] | Ireland (Dublin) | 12 publicly funded outpatient physiotherapy clinics | Baseline (255)  Week 1 (196)  Week 4 (171)  Week 12 (173)  Week 24 (207) | Control:  46.71 (±13.48); CONNECT:  44.11 (±12.96) | 54% : 46% | 1) Patient’s beliefs;  3) Patient-practitioner relationship | - CONNECT: *M* = 3.08 individual / one-on-one sessions with physical therapist (S.D. = ± 1.88) - Duration: *M* = 7.46 weeks |

**Table S1 continued.** Summary of study characteristics clustered by research design

| **Reference** | **Country of Origin** | **Study Setting** | **Total Sample size (*n*)** | **Mean age**  **Years** (*SD*) | **Gender Proportions**  (F : M %) | **Contextual Factor(s)** | **Frequency / Duration of Contextual Factor Treatment** |
| --- | --- | --- | --- | --- | --- | --- | --- |
| **Randomised Controlled Trials** (RCTs) | | | | | | | |
| [42] | Scotland | “Back to Fitness exercise classes” in the Greater Glasgow National Health Service (NHS) | Allocated (38)  Baseline (38)  Post-Tx (34)  Follow-up (27) | ED-EX:  45.5 (±9.5)  ED:  45.2 (±11.9) | 66% : 34% | 1) Patient’s beliefs;  3) Patient-practitioner relationship | - ED-EX: One 2.5-hour PNE session and 6 weekly exercise sessions (± 1 hour each) - ED: One 2.5-hour pain (neuro) biology education (PNE) session (unspecified if ED was group-based or one-on-one) |
| [43] | Japan | Single tertiary medical centre | Allocated (52)  Baseline (52)  Post-Tx (52)  Follow-up (48) | Combined:  66.8 (±13.4) | 61.5%: 38.5% | 1) Patient’s beliefs;  5) Treatment characteristics | - 12 weeks (2 capsules taken twice a day (a.m. and p.m.); - At baseline, standardised information about placebo effects (covering 5 main points) lasting (± 60 minutes. |
| [44] | Brazil | An outpatient clinic with patients from the waiting list of two University physical therapy services | Allocated (222)  Baseline (222)  Post-Tx (205)  6-months (194)  12-months (191) | ED+TA:  46.0 (± 14.7)  ED only:  47.2 (± 14.8)  No ED:  50.8 (± 13.2) | 57% : 43% | 1) Patient’s beliefs;  3) Patient-practitioner relationship | - Two individual / one-on-one sessions (± 60 minutes each) - 1-week interval between sessions |
| [45] | Germany | Department of Orthopaedics (University Hospital) | Allocated (Unclear)  Baseline (48)  Post-Tx _T1_ (48)  Post-Tx _T2_ (48) | Combined:  49.97 (±13.64) | 75% : 25% | 1) Patient’s beliefs;  5) Treatment characteristics | - One individual / one-on-one session (± 2 hours) |
| [46] | Germany | Treatment room in the Pain Therapy Section of the Department of Anaesthesiology | Allocated (96)  Baseline (85)  Post-Tx (73) | Combined:  50.04 (±11.07) | 56% : 44% | 1) Patient’s beliefs;  5) Treatment characteristics | - Two individual / one-on-one sessions (± 2 hours each). - 8-day interval between sessions |
| **Controlled Clinical Trials** (CCT; non-randomised) | | | | | | | |
| [47] | Israel | 8 participating physical therapy clinics (Public Health Services) | Baseline (220)  Post-Tx (198)  Follow-up (189) | Control:  42.0 (±7.0)  ETMI:  42.0 (±8.0) | 54% : 46% | 1) Patient’s beliefs;  3) Patient-practitioner relationship | - At least two individual / one-on-one treatment sessions (±20-30 minutes) and no upper limit. - ETMI: *M* = 3.5 sessions (S.D. = ± 1.9) |

**Table S1 continued.** Summary of study characteristics clustered by research design

| **Reference** | **Country of Origin** | **Study Setting** | **Total Sample size (*n*)** | **Mean age**  **Years** (*SD*) | **Gender Proportions**  (F : M %) | **Contextual Factor(s)** | **Frequency / Duration of Contextual Factor Treatment** |
| --- | --- | --- | --- | --- | --- | --- | --- |
| **Controlled Clinical Trials** (CCT; non-randomised) | | | | | | | |
| [48] | Germany | 4 inpatient MSK rehabilitation centres | Baseline (210)  Post-Tx (201) | Control:  54.01 (±10.99) Intervention:  54.17 (±11.82) | 64% : 36%  (11 missing) | 1) Patient’s beliefs | - Three individual / one-on-one sessions (20 minutes each) |
| [49] | Brazil | University affiliated Spine Clinic of Sports Injury Centre | Baseline (30)  Midpoint (30)  Post-Tx (30) | Not reported | 40% : 60% | 3) Patient-practitioner relationship;  4) Therapeutic setting / environment | - Rehab: *M* = 13.6 sessions (of 16; in 8 weeks) in a group-format - Home: *M* = 13.9 sessions (of 24; in 8 weeks) |
| **Quasi-experimental** (uncontrolled) | | | | | | | |
| [50] | Ireland  (Limerick) | Outpatient University affiliated treatment setting | Baseline_1 to 3_ (26)  Post-Tx (24)  Follow-up_1_ (23)  Follow-up_2_ (22)  Follow-up_3_ (21) | Combined:  44.3 (±9.73) | 54% : 46% | 1) Patient’s beliefs | - *M* = 7.7 individual / one-on-one sessions (S.D. = ± 2.5) - ± 60.0 minutes each - Duration: *M* = 12.0 weeks   (S.D. = ± 3.5) |
| [51] | United States of America (USA) | 4 physiotherapy clinics (in 2 different States) | Baseline (50)  Post-Tx (50) | Combined: 64.3 (±10.73) | 56% : 44% | 1) Patient’s beliefs | - One individual / one-on-one session (± 5 minutes) - Followed by Question-and-Answer session |
| **Observational Cohort** (uncontrolled) | | | | | | | |
| [52] | Germany | 4 inpatient & 7 outpatient orthopaedic rehabilitation centres | Baseline (688)  Post-Tx (611)  Follow-up (468) | Combined:  51.0 (±11.2) | 57% : 43% | 3) Patient-practitioner relationship | - 4–5 therapy sessions per day (on weekdays). - *M* = 20.6 days (S.D. = ± 4.5) - Unclear whether individual or group-based treatment. |
| [53] | Australia | 3 outpatient public hospital physiotherapy departments | Baseline (240)  Post-Tx (182) | Group A:  54.2 (±15.4)  Group B:  52.0 (±15.7);  Group C:  53.6 (±14.3) | 69% : 31% | 3) Patient-practitioner relationship | - Up to 12 treatment sessions - Physical therapists chose the dose and techniques based on participant’s clinical features. - Duration: 8 weeks - Exercises involved a group-format whilst those receiving spinal manipulative therapy involved individual sessions. |

**Table S1 continued.** Summary of study characteristics clustered by research design

| **Reference** | **Country of Origin** | **Study Setting** | **Total Sample size (*n*)** | **Mean age**  **Years (***SD***)** | **Gender Proportions**  (F : M %) | **Contextual Factor(s)** | **Frequency / Duration of Contextual Factor Treatment** |
| --- | --- | --- | --- | --- | --- | --- | --- |
| **Observational Cohort** (uncontrolled) | | | | | | | |
| [54] | Netherlands | Outpatient rehabilitation centre | Baseline (156)  Post-Tx (135) | Combined:  46.12 (±12.30) | 56% : 44% | 1) Patient’s beliefs | - 10-14 individual / one-on-one sessions (± 60 minutes each) |
| [55] | Not specified (possibly Australia) | University outpatient physical therapy clinic | Baseline (136)  Post-Tx (64) | Combined:  41.5 (±16.3) | 69% : 31% | 1) Patient’s beliefs | - Physical Therapy (no additional information provided) |
| **Notes:**  Post-Tx: post-treatment | | | | | | | |

**Table S2.** Contextual Factor intervention group(s) within-group change in outcomes from baseline clustered by research design

| **Ref No. (Year) & Study Design** | **Quality Assessment Grading** | **Type of Contextual Factor(s)** | **Main CF-intervention Elements** | **Mean Change (**Δ) **from Baseline:**  **Pain Intensity** | **Mean Change (**Δ) **from Baseline:**  **Physical Functioning** |
| --- | --- | --- | --- | --- | --- |
| **[35]** (2011)  RCT | Excellent  (92.9%) | 1) Patient’s beliefs;  3) Patient-practitioner relationship;  5) Treatment characteristics | **MET**: Motivational Enhancement Treatment – proxy efficacy, treatment expectancy, therapeutic alliance, and empathy, combined with conventional physical therapy. | **Significant** (***p* < .001**)  VAS (0-10)  *Post-treatment (1 month)*  ***M*Δ = 2.2**; *n* = 38 | **Significant** (**< .001**)  RMDQ (0-24)  *Post-treatment (1 month)*  ***M*Δ = 4.4**; *n* = 38 |
| **[36]** (2013)  RCT | Excellent  (89.3%) | 1) Patient’s beliefs | **CFT**: Cognitive Functional Therapy – strong focus on reframing back pain in a person-centred manner along with altering maladaptive / unhelpful behaviours to normalise movement. | **Significant** (***p* < .001**)  NRS (0-10)  *Post-treatment (3 months)*  ***M*Δ = 3.2**; *n* = 51;  95% C.I. [2.5 – 3.9] | **Significant** (***p* < .001**)  ODI (0-100)  *Post-treatment (3 months)*  ***M*Δ = 13.7**; *n* = 51  95% C.I. [11.4 – 16.1] |
| **[37]** (2014)  RCT  (2x2) | Excellent  (89.3%) | 3) Patient-practitioner relationship;  5) Treatment characteristics | Enhanced Therapeutic Alliance (**TA**) versus limited TA in patients receiving either active or sham interferential current therapy (IFC). | **Clinically important**  NRS (0-10)  *Post-treatment (1 session)*  **Enhanced Therapeutic Alliance**  Active IFC: ***M*Δ = 3.13**; *n* = 29  77.4% ↓ in pain intensity  Sham IFC: ***M*Δ = 2.22**; *n* = 29  54.5% ↓ in pain intensity | *Not applicable* |
| **[38]** (2022)  RCT | Excellent  (89.3%) | 1) Patient’s beliefs | **PRT**: Pain Reprocessing Therapy – aims to shift patients’ beliefs about the causes and threat value of pain via five main components: 1) education about the brain origins and reversibility of pain; 2) reinforcing personalised evidence for (1) above, 3) attending to and appraising pain sensations through a safety lens; 4) addressing other emotional threats; and 5) gravitating to positive feelings and sensations. | **Clinically important**  NRS (0-10)  *Post-treatment (4 weeks)*  ***M*Δ = 3.04**; *n* = 44  78% of patients: 30% pain reduction  70% of patients: 50% pain reduction  66% of patients: nearly pain-free  *Follow-up (1-year)*  ***M*Δ = 2.71**; *n* = 45  70% of patients: 30% pain reduction  60% of patients: 50% pain reduction  52% of patients: nearly pain-free | **Improvement**  *p-value unknown*  ODI (0-100)  *Post-treatment (4 weeks)*  ***M*Δ = 13.56**; *n* = 44  *Follow-up (1-year)*  ***M*Δ = 12.54**; *n* = 45 |

**Table S2 continued.** Contextual Factor intervention group(s) within-group change in from baseline by study design

| **Ref No. (Year) & Study Design** | **Quality Assessment Grading** | **Type of Contextual Factor(s)** | **Main CF-intervention Elements** | **Mean Change (**Δ) **from Baseline:**  **Pain Intensity** | **Mean Change (**Δ) **from Baseline:**  **Physical Functioning** |
| --- | --- | --- | --- | --- | --- |
| **[39]** (2019)  RCT | Excellent  (89.3%) | 1) Patient’s beliefs;  5) Treatment characteristics | Adding open-label placebo (**OLP**) pills (i.e., response expectancy & labelling) using social learning (video) and verbal suggestions to treatment as usual (TAU). | **Improvement**  *p-value unknown*  NRS (0-10)  *Post-treatment (3 weeks)*  ***M*Δ = -0.62**; *n* = 63 | **Improvement**  *p-value unknown*  ODI (0-100) / PSFS (0-10)  *Post-treatment (3 weeks)*  **ODI: *M*Δ = -3.21**; *n* = 63  **PSFS: *M*Δ = 0.94**; *n* = 63 |
| **[40]** (2016)  RCT | Excellent  (85.7%) | 1) Patient’s beliefs;  5) Treatment characteristics | Adding open-label placebo (**OLP**) pills (i.e., response expectancy & labelling) to treatment as usual (TAU), along with verbal suggestions (scripted dialogue) & social learning (video). | **28**% **Improvement**  *p-value unknown*  NRS (0-10)  *Post-treatment (3 weeks)*  ***M*Δ = 1.49**; *n* = 41 | **29**% **Improvement**  *p-value unknown*  RMDQ (0-24)  *Post-treatment (3 weeks)*  ***M*Δ = 2.86**; *n* = 41 |
| **[41]** (2017)  RCT  (Cluster) | Excellent  (85.7%) | 1) Patient’s beliefs;  3) Patient-practitioner relationship | **CONNECT**: Enhancing physiotherapists’ need-supportive communication skills to address maladaptive / unhelpful patient beliefs and improve motivation. | **Improvement**  *p-value unknown*  NRS (0-10)  *Post: (12 weeks) / Follow-up (24 weeks)*  ***M*Δ = -1.53 / *M*Δ = -1.53**; *n* = 108 | **Improvement**  *p-value unknown*  RMDQ (0-24)  *Post: (12 weeks) / Follow-up (24 weeks)*  ***M*Δ = -3.48 / *M*Δ = -4.87**; *n* = 108 |
| **[42]** (2010)  RCT | Good (82.1%) | 1) Patient’s beliefs;  3) Patient-practitioner relationship | Pain (neuro) biology education for the management of cLBP with and without group-exercise classes. | **Improvement**  *p-value unknown*  NRS (0-100)  *Post / Follow-up (12 weeks)*  ***M*Δ = 30.9 / *M*Δ = -16.7**; *n* = 16 | **Improvement**  *p-value unknown*  RMDQ (0-24)  *Post / Follow-up (12 weeks)*  ***M*Δ = -7.5 / *M*Δ = -6.5**; *n* = 16 |
| **[43]** (2020)  RCT | Good (82.1%) | 1) Patient’s beliefs;  5) Treatment characteristics | Adding open-label placebo (**OLP**) pills (i.e., response expectancy) using verbal suggestions (scripted dialogue) to treatment as usual (TAU). | **Not Significant** (*p* = *.*17)  NRS (0-10)  *Post-treatment (3 weeks)*  ***M*Δ = -0.9**; *n* = 26  5 patients (19.2%) met or surpassed the MCID (≥ 2-unit change from baseline).  *Follow-up (12 weeks)*  ***M*Δ = -1.1**; *n* = 24  11 patients (45.8%) met or surpassed the MCID (≥ 2-unit change from baseline). | **RMDQ Significant** (**< .01**)*  RMDQ (0-24)  **TUG Not Significant** (*p* = *.*10)  TUG (seconds)  *Post-treatment (3 weeks)*  ***RMDQ**: ***M*Δ = -2.2**; *n* = 26  **TUG**: ***M*Δ = -0.7**; *n* = 26  *Follow-up (12 weeks)*  ***RMDQ: *M*Δ = -3.3**; *n* = 24  **TUG**: ***M*Δ = -0.6**; *n* = 24 |
| **Notes:**  PSFS: Patient-Specific Functional Scale – higher scores represent higher levels of functioning; MCID: minimal clinically important difference; TUG: Timed-Up-and-Go (measured in seconds) | | | | | |

**Table S2 continued.** Contextual Factor intervention group(s) within-group change in from baseline by study design

| **Ref No. (Year) & Study Design** | **Quality Assessment Grading** | **Type of Contextual Factor(s)** | **Main CF-intervention Elements** | **Mean Change (**Δ) **from Baseline:**  **Pain Intensity** | **Mean Change (**Δ) **from Baseline:**  **Physical Functioning** |
| --- | --- | --- | --- | --- | --- |
| **[44]** (2021)  RCT | Good (82.1%) | 1) Patient’s beliefs;  3) Patient-practitioner relationship | Patient education (ED) relating to return to daily activities, advice on coping with pain, a clear explanation of signs and symptoms with an emphasis on increasing empathy and therapeutic alliance (TA) in one treatment group (ED+TA). | **Improvement**  *p-value unknown*  NRS (0-10)  *Post-treatment (1-month)*  **ED+TA: *M*Δ = 2.1**; *n* = 68  **ED only: *M*Δ = 2.2**; *n* = 69  *Follow-up (6-months)*  **ED+TA: *M*Δ = 1.62**; *n* = 65  **ED only: *M*Δ = 2.33**; *n* = 65  *Follow-up (1-year)*  **ED+TA: *M*Δ = 2.53**; *n* = 64  **ED only: *M*Δ = 2.62**; *n* = 65 | **Improvement**  *p-value unknown*  ODI (0-100) / PSFS (0-10)  *Post-treatment (1-month)*  **ED+TA: *M*Δ ODI = 6.26 / PSFS** = **1.9**  **ED only: *M*Δ ODI = 5.12 / PSFS = 1.49**  *Follow-up (6-months)*  **ED+TA: *M*Δ ODI = 7.78 / PSFS** = **1.66**  **ED only: *M*Δ ODI = 7.17** / **PSFS** = **1.31**  *Follow-up (1-year)*  **ED+TA: *M*Δ ODI = 11.54 / PSFS** = **2.13**  **ED only: *M*Δ ODI = 9.92 / PSFS** **= 1.88** |
| **[45]** (2017)  RCT  (2x2) | Good  (78.6%) | 1) Patient’s beliefs;  5) Treatment characteristics | Manipulating patient’s expectations using an inert solution / labelling, verbal instructions, with or without classical conditioning (CC). | **Significant** (***p* < .001**)*****  NRS (0-10)  *Post-treatment (same day)*  Opioid Instruction (*n* = 24)  *With CC: ***M*Δ = 3.16**  *No CC: ***M*Δ = 2.00**  Placebo Instruction (*n* = 24)  With CC: ***M*Δ = 0.67; (***p* < 0.26)  *No CC: ***M*Δ = -1.16*** *(increased pain)* | **Significant** (***p* < .001**)*****  ADL (0-100%)  *Post-treatment (same day)*  Opioid Instruction (*n* = 24)  *With CC: ***M*Δ = -16.66**  *No CC: ***M*Δ = -15.26**  Placebo Instruction (*n* = 24)  With CC: ***M*Δ = -3.89**; *p* =.22  No CC: ***M*Δ = 6.67**; *p* = .06 |
| **[46]** (2019)  RCT  (2x2) | Good  (71.4%) | 1) Patient’s beliefs;  5) Treatment characteristics | Manipulating patient’s expectations using a sham “opioid” infusion with mirrors / labelling, verbal instructions, and either placebo or nocebo conditioning (PC or NC). Sham “Opioid” Infusion:  Placebo (**PC**): (*n* = 17)  Sham only (**SO**): (*n* = 21)  Nocebo (**NC**): ( *n*= 21)  Natural History  (**NH**): (*n* = 14) | **Significant** (***p* < .001**)*****  NRS (0-10)  *Post-treatment (Day 1 / Day 8)*  Sham “Opioid” Infusion:  ***PC: *M*Δ = 2.23 /** Day 8 ***M*Δ = 2.06**  ***SO: *M*Δ = 3.14 /** Day 8 ***M*Δ = 3.00**  ***NC: *M*Δ = 1.48 /** Day 8 ***M*Δ = 1.57**  Natural History (*p* = 0.92)  NH: ***M*Δ = 0.29 /** Day 8 ***M*Δ = -0.07** | **Significant** (***p* < .001**)*****  ADL (0-100%)  *Post-treatment (Day 1 / Day 8)*  Sham “Opioid” Infusion:  ***PC: *M*Δ = 5.09 /** Day 8 ***M*Δ = 9.41**  ***SO: *M*Δ = 12.7 /** Day 8 ***M*Δ = 13.97**  ***NC: *M*Δ = 12.07 /** Day 8 ***M*Δ = 17.47**  Natural History (*p* = 0.63)  NH: ***M*Δ = 1.43 /** Day 8 ***M*Δ = 0.53** |
| **Notes:**  ADL: Patient-Specific Functional Scale – higher scores represent higher levels of functioning; ADL: Hannover Activities of Daily Living Questionnaire. | | | | | |

**Table S2 continued.** Contextual Factor intervention group(s) within-group change in from baseline by study design

| **Ref No. (Year) & Study Design** | **Quality Assessment Grading** | **Type of Contextual Factor(s)** | **Main CF-intervention Elements** | **Mean Change (**Δ) **from Baseline:**  **Pain Intensity** | **Mean Change (**Δ) **from Baseline:**  **Physical Functioning** | |
| --- | --- | --- | --- | --- | --- | --- |
| **[47]** (2017)  CCT | Excellent  (88.5%) | 1) Patient’s beliefs;  3) Patient-practitioner relationship | **ETMI (**Enhanced Transtheoretical Model Intervention) targeted cLBP intervention focusing on therapists’ communication skills, therapeutic alliance, low motivation, patient’s self-efficacy, and maladaptive or unhelpful beliefs/behaviour. | **Improvement**  *p-value unknown*  NRS (0-10)  Average Pain (*n* = 94)  *3-months:* ***M*Δ = 2.1** 95% C.I. [1.5-2.7]  *1 year:* ***M*Δ = 2.6** 95% C.I. [1.9-3.3]  Worst Pain (*n* = 94)  *3-months:* ***M*Δ = 1.9** 95% C.I. [1.2-2.7]  *1 year:* ***M*Δ = 2.9** 95% C.I. [2.0-3.7] | **Improvement**  *p-value unknown*  RMDQ (0-23)  *3-months:* ***M*Δ = 4.9** 95% C.I. [3.7-6.1]  *1 year:* ***M*Δ = 6.7** 95% C.I. [5.4-8.0]  84% achieved ≥ 30% reduction in physical disability (*n* = 94). | |
| **[48]** (2012)  CCT | Good  (73.1%) | 1) Patient’s beliefs | Targeted cLBP intervention focusing on patient’s illness and treatment beliefs along with their individual information needs. | **Significant** (***p* < .001**)  VAS (0–100)  *Post-treatment*  ***M*Δ = -14.91**; *n* = 93;  Standardised Effect Size = -0.66 | **Significant (*p* = .002)**  ODI (0–100)  *Post-treatment*  ***M*Δ = -3.26**; *n* = 92  Standardised Effect Size = -0.16 | |
| **[49]** (2018)  CCT | Fair  (65.4%) | 3) Patient-practitioner relationship;  4) Therapeutic setting / environment | Adding one weekly group-based physical therapy session in a rehabilitation setting compared to home treatment alone. | **Significant** (***p* < .001**)  NRS (0-10)  *Post-treatment (8 weeks)*  Rehab: ***M*Δ = 2.0**, *n* = 13 | **Significant** (***p* < .001**)  RMDQ (0-24)  *Post-treatment (8 weeks)*  Rehab: ***M*Δ = 2.8**, *n* = 13 | |
| **[50]** (2015)  Quasi-exp.  (*Interrupted Time Series*) | Excellent  (86.4%) | 1) Patient’s beliefs | **CFT**: Cognitive Functional Therapy – strong focus on reframing back pain in a person-centred manner along with altering maladaptive / unhelpful behaviours to normalise movement. | **Significant** (***p* < .001**)  NRS (0–10)  *Post:* ***M*Δ = 1.6**; *n* = 24  *1-year:* ***M*Δ = 1.7**; *n* = 21  13 patients (54.2%) met or surpassed the MCID (i.e., ≥ 30% reduction at 12-months) | **Significant** (***p* < .001**)  ODI (0–100)  *Post:* ***Median* Δ = 22**; *n* = 24  *1-year:* ***Median* Δ = 24**; *n* = 21  15 patients (62.5%) met or surpassed the MCID (i.e., ≥ 30% reduction at 12-months) | |
| **[51]** (2017)  Quasi-exp.  (*Case Series*) | Good  (81.8%) | 1) Patient’s beliefs | Pain (neuro)science education (PNE) focusing on altering beliefs regarding cLBP and aging. | **Significant** (***p* = .002**)  NRS (0–10)  *Post-treatment (same day)*  ***M*Δ = −0.5**; *n* = 50; *r* = 0.45  21 patients (42%) met or surpassed the MCID (≥ 1-unit change). | **Significant** (***p* < .001**)  Active trunk forward flexion (cm)  *Post-treatment (same day)*  ***M*Δ = −4.0cm**; *n* = 50  18 patients (36%) met or exceeded the MCID (≥ 4.5cm) | |
| **Notes:**  MCID: minimal clinically important difference; *post-tx*: post-treatment | | | | | |  |

**Table S2 continued.** Contextual Factor intervention group(s) within-group change in from baseline by study design

| **Ref No. (Year) & Study Design** | **Quality Assessment Grading** | **Type of Contextual Factor(s)** | **Main CF-intervention Elements** | **Mean Change (**Δ) **from Baseline:**  **Pain Intensity** | **Mean Change (**Δ) **from Baseline:**  **Physical Functioning** |
| --- | --- | --- | --- | --- | --- |
| **[52]** (2013)  Obs. Cohort | Excellent  (95.5%) | 3) Patient-practitioner relationship | *No manipulation* - measuring pre-existing relational aspects: -perceived involvement in care; trust; satisfaction; healthcare practitioner’s communication behaviour. | **Significant** (***p* < .001**)  VAS (0–100)  *Post-treatment*  ***M*Δ = 11.54**; *n* = 611  *Follow-up (6-months)*  ***M*Δ = 12.67** *n* = 468 | **Significant** (***p* < .001**)  ODI (0–100)  *Post-treatment*  ***M*Δ = 4.96**; *n* = 599  *Follow-up: (6-months)*  ***M*Δ =**  **7.21**; *n* = 468 |
| **[53]** (2013)  Obs. Cohort | Excellent  (90.9%) | 3) Patient-practitioner relationship | *No manipulation* - measuring pre-existing relational aspects: - therapeutic alliance (**TA**) between patients and their practitioner (scale range 16-112). | **Significant (*p* = .001)**  VAS (0–10)  **↑ TA ↓ pain**  β = -0.044, *n* = 182  One unit increase in TA reduced pain by 0.044 units. | **Significant (*p* < .000)**  RMDQ (0-24)  **↑ TA ↓ physical disability**  β = –0.113, *n* = 182  One unit increase in TA reduced disability by 0.113 units. |
| **[54]** (2011)  Obs. Cohort | Excellent  (86.4%) | 1) Patient’s beliefs | Targeted cLBP intervention focusing on addressing maladaptive illness perceptions (predictors: rational problem-solving, discussion skills, and verbal skills) via Socratic dialogue. | *Not applicable* | **Significant (*p* = .014)**  PSFS (0–100)  **↑ Rational Problem-Solving (RPS)**  **↓ physical disability**  β = –0.49, *n* = 136; *r^2^ =* 3.9%  One unit increase in the RPS scale results in a decrease of 0.49 in the Patient-Specific Functioning Scale (PSFS). |
| **[55]** (2019)  Obs. Cohort | Excellent  (86.4%) | 1) Patient’s beliefs and characteristics | *No manipulation –* measuring patient’s competence perceptions (CP) to perform required physical therapy tasks and their self-reported motivations for undertaking physical therapy where, *amotivation* represents the least self-determined type and *autonomous* motivation is the most self-determined along a continuum. | **Significant** (***p* < .01**)  NRS (0–10)  **↑ CP ↓ pain**  ***r* = –0.34** (negative, moderate correlation with CP) (*n* = 64)  **↑ amotivation ↑ pain**  ***r* = 0.48** (positive, moderate correlation with amotivation)  The individual indirect effect of amotivation on pain was statistically significant (*p* < .05). Thus, lower perceptions of competence were predictive of stronger amotivation, which was in turn predictive of greater pain. The percent of the pain model mediated through amotivation was 44.7%. | **Significant** (***p* < .01**)  ODI (0–100)  **↑ CP ↓ physical disability**  ***r* = –0.35** (negative, moderate correlation with CP) (*n* = 64)  **↑ amotivation ↑ physical disability**  ***r* = 0.39** (positive, moderate correlation with amotivation)  The individual indirect effect of amotivation on disability was statistically significant (*p* < .05). Thus, lower perceptions of competence were predictive of stronger amotivation, which was in turn predictive of greater disability levels. The percent of the disability model mediated through amotivation was 70.2%. |
